# Factors associated with stunting among children 0 to 59 months of age in Angola: A cross-sectional study using the 2015–2016 Demographic and Health Survey

**DOI:** 10.1371/journal.pgph.0000983

**Published:** 2022-12-12

**Authors:** Paulo Renato Correa

**Affiliations:** Programme in Epidemiology, London School of Hygiene and Tropical Medicine, London, United Kingdom; Translational Health Science and Technology Institute, INDIA

## Abstract

Stunting among children under five years of age is a serious public health problem globally, with life-long consequences to health, well-being, and productivity. Stunted growth has complex and multifactorial causes, reflecting the interaction of a broad range of conditions that determine child health. The Angola 2015–2016 Demographic and Health Survey (DHS) collected nationally representative anthropometry for 6,359 children 0 to 59 months of age in Angola, and ascertained exposure to a wide range of child, parental, socio-economic, and geographic variables. This study used a cross-sectional design to identify exposures associated with stunting among children 0 to 59 months of age in Angola, while considering the multifactorial and multi-level causes of stunting. Main outcome was prevalence of stunting, defined as proportion of children with height-for-age Z-score (HAZ) two or more standard deviations below the median. Prevalence of stunting was associated with individual, household, and area-level exposure variables, including child age and sex, birth order, birthweight, diarrhea, maternal and paternal age and education, source of water, sanitary system, and province. In conclusion, prevalence of stunting in Angola is associated with several factors previously described in the literature. Stunting is associated with exposures at the distal, intermediate, and proximal levels, in line with the framework on the causes of childhood malnutrition. This study identifies opportunities for interventions at multiple levels to decrease prevalence of stunting among children in Angola. Main limitations of this study are the potential for survival bias and residual confounding.

## Introduction

Stunted growth remains a major public health problem globally, with one in five children suffering from stunting before they reach five years of age [1]. Stunting is particularly common in low-income countries (LICs), where the prevalence was estimated at 34.6% [1]. In Africa, prevalence decreased from 41.5%, in 2000, to 30.7% in 2020, but the absolute number of cases increased, from 54.4 million, in 2000, to 61.4 million in 2020 [1]. In Angola, prevalence of stunting was recently estimated at 37.7% among children 0 to 59 months of age, but those estimates were not based on a nationally representative survey [1].

Stunted growth is defined as length- or height-for-age two or more standard deviation (SD) below the expected median (height-for-age Z-score, HAZ ≤ -2), based on the 2006 World Health Organization (WHO) Child Growth Standards [2, 3]. Stunting results from the interaction of multiple risk factors described in the conceptual framework *“Childhood stunting*: *Context*, *Causes*, *and Consequences*” [4], with exposures operating at three levels: proximal, intermediate, and distal [5]. Stunted growth disrupts normal child development and has life-long consequences to health and productivity of individuals [6–8], resulting in cognitive [9, 10] and psychological deficits [11, 12], and increasing the risk of chronic diseases of adulthood [7, 13]. Stunted growth also has a transgenerational effect, impacting the health of children born to the generation exposed to malnutrition and stunting [14]. Consequently, stunting of growth during childhood represents a major challenge for development in low- and middle-income countries (LMICs), including those in the African continent [15, 16]. Given the importance of stunting for present and future generations, the United Nations Sustainable Development Goals (SDG) includes the goal to “end all forms of malnutrition, including achieving, by 2025, the internationally agreed targets on stunting and wasting in children under 5 years of age (…)”. The current WHO and UNICEF target is a 50% reduction in prevalence of stunting by 2030, taking 2012-level as the baseline [17]. However, prevalence of stunting is not decreasing as rapidly as needed to meet those targets. At the current pace, only a quarter of the countries will meet goals for reduction in prevalence of stunting [1, 18]. Consequently, there is an urgent need to obtain current estimates for the prevalence of stunting and identify risk factors in vulnerable countries.

The public health impact of childhood malnutrition and stunting is of particular concern for countries in southern Africa, where prevalence remains elevated despite recent socioeconomic transformation in the region [19, 20]. Angola is an example of this development paradox. Emerging from a protracted civil war, following centuries of colonization, Angola experienced a rapid transition into a free market economy, driven by exploration of oil and gas [21]. However, Angola continues to display marked socioeconomic inequalities, and some of the worst indicators of poverty, infrastructure, education and health [22]. One major gap in public health in Angola was the lack of information on prevalence and risk-factors for stunting. The Angola 2015–2016 Demographic and Health Survey (DHS) was the first survey to collected nationally representative data on child anthropometry and a range of individual, household, and area level exposures [23]. The aim of this study was to use data from the Angola 2015–2016 DHS to describe the frequency and distribution of stunted growth according to a comprehensive set of exposure variables and to identify factors associated with stunting among children 0 to 59 months of age.

## Methods

### Study design

This is a cross-sectional study using data from a population-based survey of households. The Angola Demographic and Health Survey 2015–2016 (Inquérito de Indicadores Múltiplos e de Saúde, IIMS 2015–2016) [23] was implemented by the Angola National Institute of Statistics (INE) in collaboration with the Ministry of Health of Angola (MINSA) and Ministry of Planning and Territorial Development (MPDT). Technical assistance was provided by UNICEF and ICF, through the DHS Program, with logistical support from WHO. Survey was funded by the United States Agency for International Development (USAID), The World Bank, UNICEF, and the Government of Angola. The IIMS 2015–2016 is a population-based, nationally representative, cross-sectional survey of households [23]. Data used in this project is freely and publicly available from The DHS Program website, and does not contain any identifiable information [24]. Methods used in data collection and anthropometric measurements are described in the DHS Interviewer’s Manual [25]. Prior to deployment of survey at a national level, a pilot survey was completed in Luanda to evaluate and validate the training of staff in the administration of questionnaires, and performance of questionnaires in the field. The staff conducting field survey in the 18 provinces was trained together by staff of INE and ICF over a period of six weeks through theoretical classes and practical exercises. To ensure the quality and consistency of measurement of height and weight, survey technicians received training on proper techniques to perform anthropometry [23].

### Ethics statement

Ethics review of the Angola DHS 2015–2016 (ICF Project Number: 132989.0.000) was conducted by the ICF Institutional Review Board (IRB) which approved the research protocol (ICF IRB FWA00000845). ICF IRB attested that the protocol complies with all the requirements of the title 45, Public Welfare, Code of Federal Regulations, Part 46, Protection of Human Subjects (45 CFR Part 46). Authorization for data usage was granted by ICF (9300 Lee Highway, Fairfax, VA 22031, USA). Participation was voluntary and written informed consent was obtained from all participants. Separate written informed consent was obtained for each portion of the survey: household, women, and men questionnaires, biometric sample collection, and anthropometry. In the case of children, written informed consent was obtained from parents or legal guardians. Written informed consent forms are available in the final report [24].

### Setting and participants

**Fig 1:**
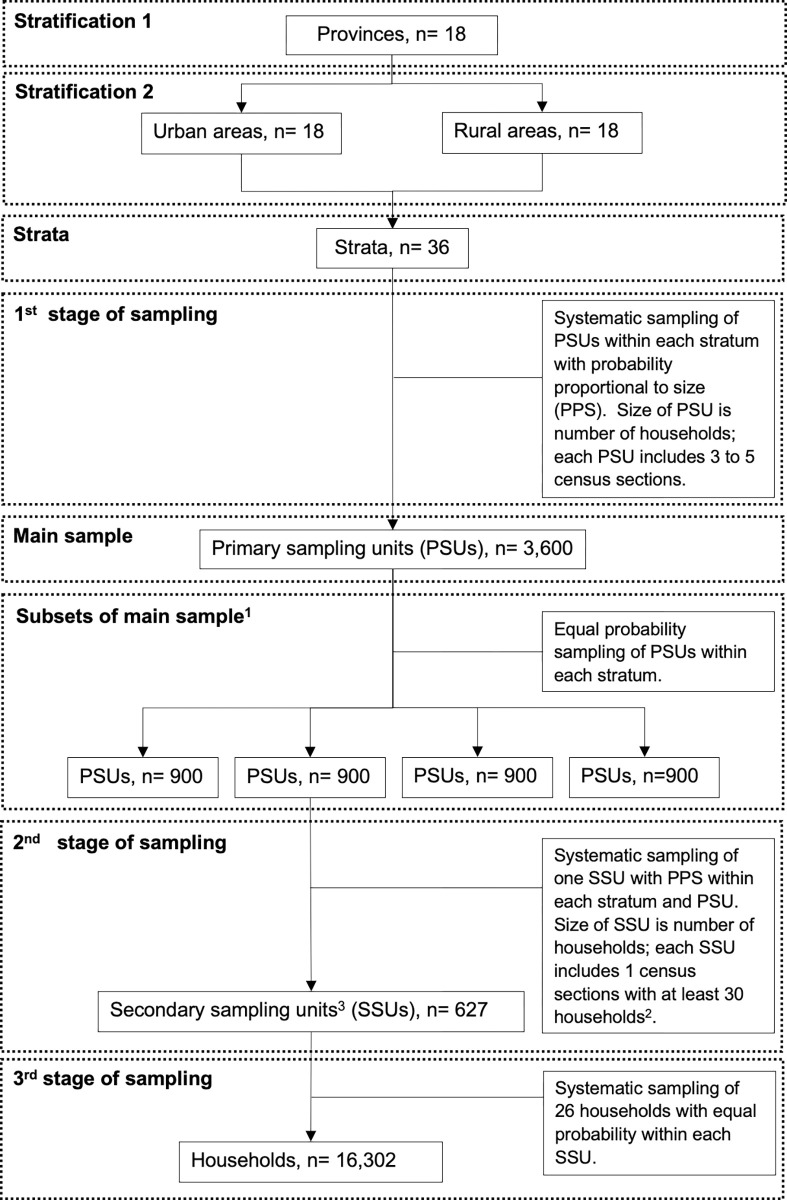
Sampling procedure used in the Angola 2015–2016 Demographic and Health Survey (DHS). Flowchart describing sampling procedure, including stratification, stages of selection of sampling units, methods of sampling used, and number of households selected. ^1^Each subset of the primary sample is equally representative of the main sample and of the country population. ^2^ Census sections have an average of 83 households (103 in urban areas, and 63 in rural areas). Census sections with less than 30 households were combined to form secondary sampling units (SSUs) with at least 30 households. ^3^ Sampling resulted in 33 SSUs per province, except Luanda, where 66 SSUs were sampled; 345 SSUs were in urban areas, and 282 in rural areas.

Households in urban and rural areas of the 18 provinces of the Republic of Angola (República de Angola) were selected for participation and surveyed between November, 2015 and February, 2016 [23]. Sample of household surveyed was obtained using a multi-stage, stratified, probabilistic, systematic, and clustered sampling procedure, following DHS methodology [26], as described in the IIMS 2015–2016 report [23]. The Angola 2014 census (Recenseamento Geral da População e Habitação, RGPH), provided the sampling frame for selection of primary sampling units (PSUs). Selection of PSUs was stratified by the 18 provinces of Angola and rural/urban areas to ensure representation, resulting in 36 strata (Fig 1). In the first stage, primary sampling units (PSUs), defined as groups of three to five census sections, were selected systematically within each stratum with probability proportional to number of households in each PSU (probability proportional to size, PPS) (Fig 1). The first stage of sampling generated 3,600 PSUs, which were divided in four equally representative replication samples, with 900 PSUs each (Fig 1). The second stage consisted of selecting one secondary sampling unit (SSU) within each PSU using systematic sampling with probability proportional to size (PPS). A total of 627 SSUs (clusters of households) were selected. Each SSU consisted of a census section with at least 30 households. Census sections with less than 30 households were aggregate before sampling of SSUs. The third stage of sampling consisted of selecting 26 households within each SSUs using systematic sampling with equal probability. The sampling procedure was designed to obtain a nationally representative sample of 16,302 households. A total of 16,244 households were visited in the survey [23] (S1 Fig). Women 15 to 49 years of age and their children 0 to 59 months of age who slept in the selected households the previous night were eligible for inclusion in the survey. Participation was voluntary and informed consent obtained according to Good Clinical Research Practice. Privacy and confidentiality of participant data was ensured during data collection, processing, and analysis. Details of the number of households visited, consented for participation, women screened for eligibility and included in the study is available in the S1 Fig.

### Exposures and outcome

Exposures available in the Angola 2015–2016 dataset were selected for inclusion based on risk factors for stunting described in the literature [27, 28] and according to the current conceptual framework for the causation of stunting [4, 29]. Information on exposures was ascertained in direct interviews using questionnaire validated in prior DHS surveys [23, 30]. Characteristics of children, parents, and households, including socioeconomic and geographic variables, were included in the analysis. Characteristics of children included sex of child, child age, birth order, birth weight, newborn health visit, and duration of breast feeding. Maternal characteristics included age-group, cohabitation status, educational level, age at sexual initiation, measure of sexual autonomy (woman can refuse sex) and safe sex autonomy (woman can ask partner to use a condom), life-time use of natality control, autonomy to visit family (who decides to visit family) and to make healthcare decisions (who makes healthcare-related decisions), number of antenatal care (ANC) visits, and work outside of home. Paternal characteristics included age-group and educational level Perinatal and early childcare variables included number of antenatal care (ANC) visits, newborn health visit, and duration of breast feeding. Household characteristics included both household infrastructure (source of water, sanitary system, sharing of toilet, type of floor, cooking fuel electrification of household, and refrigerator ownership) and composition (number of household members, women 15- to 49-year-old, and children under five years of age). Socioeconomic and geographic variables included wealth index, area of residence (rural and urban), and province. Primary outcome was stunted growth among children 0 to 59 months of age. Children with a height-for-age Z-score (HAZ) two, or more, standard deviations (SD) below the median in the WHO Child Growth Standards 2006 [3] were classified as stunted.

### Study size and power

Study size was determined by the size of the original survey, but sample available in the Angola 2015–2016 DHS was compared to sample size calculated to estimate the prevalence of stunting with a 2.5% margin of error. Sample size calculations assumed a relative standard error (RSE) of proportion of 0.03 [26], and an expected prevalence of 38.8% [27]. Design effects of 1.5 and 1.8 were used to account for clustering of observations [31], although there is evidence that the intracluster correlation (ICC) for stunting is small in DHS surveys [32]. Full description of sample size calculation is provided in S1 Appendix. Sample available (weighted number, n = 5,905) surpassed the sample size calculated with a design effect of 1.5 (n = 3,943) and 1.8 (n = 5,678) (S1 Appendix). Sample available in the Angola 2015–2016 DHS was also compared to sample size calculated to detect odds-ratios ranging from 0.77 to 0.91 and from 1.10 to 1.3, assuming an alpha of 0.05, power of 0.8, prevalence of 38.8% in the unexposed group, two exposure groups of equal size, and design effect of 1.5 and 1.8 (S1 Table). Sample size available (weighted number, n = 5,905) is sufficient to detect an odds-ratio (OR) equal of larger than 1.20, or equal or smaller than 0.83, for a design effect of 1.5 (S1 Table). For a design effect of 1.8, sample size available could detect an OR equal or larger than 1.25, or equal or smaller than 0.80 (S1 Table).

### Statistical methods

Statistical analysis was conducted using Stata SE 16.1 (StataCorp, College Station, Texas, 77845, USA), and considered the complex sampling procedure used in Demographic and Health Surveys [26]. Sampling weights were applied using the *svyset* command. Individual children were the unit of analysis. Complete record analysis was performed. Sample was described in terms of characteristics of children, parents, households, and socioeconomic variables by tabulating survey-weighted counts and proportions. Prevalence of stunting was estimated for overall sample and according to exposure variables. Prevalence was expressed as percentage with 95% confidence interval (95% CI). Association between exposures and stunting was tested using the Pearson’s Chi-squared test and design-based F-score. Prevalence proportion ratio (PPR) [33], also referred to as prevalence ratio (PR), was used as the primary effect measure. Stunting was expected to be a common occurrence in the population surveyed, and prevalence proportion ratio (PPR) has been shown to provide a more accurate and meaningful measure of relative risk than odds-ratio (OR) [33, 34]. Poisson regression was used to estimate crude (PR) and adjusted prevalence ratios (aPR) [35]. Effect of exposures on prevalence of stunting was also reported as absolute risk difference. Log likelihood ratio was used for hypothesis testing and to obtain P-values [36, 37]. Estimates of prevalence ratio were adjusted for confounding using multivariable Poisson regression models. Variables included in the regression models were defined *a priori* based on risk factors for stunting described in the literature [27], while considering the conceptual hierarchical framework on the causes of stunting [4, 38]. Three separate models were developed: model 1, non-modifiable exposures and exposures distal to the outcome; model 2, intermediate and modifiable exposures; and model 3, modifiable exposures proximal to outcome. Exposure variables included in each model are presented in the Results section. To detect effect modification by child age-group, stratified analysis was performed using the comprehensive multivariable model (model 3) and child age-group as the stratifying variable. Child age was categorized into three age groups: 0 to 5 months of age, 6 to 23 months of age, and 24 months of age and older. Lastly, to test if the associations were robust to adjustment for clustering of observations in primary sampling units (PSUs) and provinces, multilevel, mixed-effects, multivariable Poisson regression was used to fit a three-level random-intercept Poisson model. Random-effects were estimated for primary sampling units (PSUs) and provinces.

### Geographic Information System (GIS)

Maps were designed using QGIS version 3.16.11- Hannover [39] and shapefile for the 18 provinces of Angola was downloaded from Global Administrative Areas (GADM) website (https://gadm.org/maps/AGO.html) [40]. Terms and conditions of use available from https://gadm.org/license.html.

## Results

### Participants and descriptive data

In total, 16,244 households, located in 627 primary sampling units, were selected for participation, and 16,109 (99.2%) assessed for eligibility. A sample of 14,975 eligible women (15 to 49 years of age) was identified, and 14,379 (96%) consented to be interviewed [23] (S1 Fig). Half of the households were chosen for anthropometry, resulting in 6,765 (weighted count, n = 6,296) (47.2%) living children 0 to 59 months of age. Anthropometric data was available for 6,359 (weighted count, n = 5,905) (94%) of those children (S1 Fig). Number and proportion of children according to exposure variables and outcome are presented (Table 1). Sample of children selected (weighted count, n = 6,296) and those included in the analysis (weighted count, n = 5,905) showed similar distribution according to exposure variables and data on outcome was missing at random (S2 Table).

**Table 1:** Number of children 0 to 59 months of age according to characteristics of participants and households, and prevalence of stunting.

Characteristic	Outcome		Prevalence of stunting, % 95% CI)	p-value
	Healthy, number^1^	Stunted, number^1^		
**Total**	3,695	2,209	37.4 (35.3, 39.6)	-
**Sex**				
Female	1,959	992	33.6 (31.2, 36.2)	<0.0001
Male	1,736	1,218	41.2 (38.5, 44.1)	
**Child age-group, months**				
0 to 11	1,117	292	20.7 (17.9, 23.9)	<0.0001
12 to 23	661	554	45.6 (41.3, 49.9)	
24 to 35	574	545	48.7 (44.9,52.5)	
36 to 47	683	493	41.9 (38.1, 45.9)	
48 to 59	661	326	33.0 (29.4, 36.9)	
**Birth order**				0.1101
First	789	416	34.5 (30.9, 38.3)	
Second	774	414	34.8 (31.4, 38.5)	
Third and fourth	1,108	708	39.0 (34.8, 43.3)	
Fifth and above	1,024	671	39.6 (36.2, 43.1)	
**Birthweight (grams)**				
Low (< 2,500)	186	147	44.2 (36.8, 51.8)	<0.0001
Normal (2,500 to 3,999)	1,688	717	29.8 (26.1, 33.8)	
High (≥ 4,000)	445	153	25.6 (21.2, 30.7)	
Not weighed at birth	1,235	1,081	46.7 (44.3, 49.1)	
Missing	141	110	43.8 (35.7, 52.3)	
**Diarrhea in last 2 weeks**				
Yes	513	434	45.9 (41.3, 50.5)	<0.0001
No	3,174	1,773	35.8 (33.6, 38.1)	
Missing	9	2	19.2 (7.5, 41.0)	
**Fever in last 2 weeks**				
Yes	574	386	40.2 (35.7, 44.8)	0.2256
No	3,117	1,822	36.9 (34.5, 39.3)	
Missing	4	2	35.6 (17.5, 59.0)	
**Cough in last two weeks**				
Yes	462	276	37.4 (32.7, 42.4)	0.999
No	3,231	1,931	37.4 (35.1, 39.8)	
Missing	3	2	37.6 (14.5, 68.1)	
**Maternal age-group, years**				
15 to 19	336	197	37.0 (32.8, 41.4)	0.1575
20 to 24	941	656	41.1 (37.5, 44.7)	
25 to 29	1,003	544	35.2 (31.6, 38.9)	
30 to 34	681	383	36.0 (30.9, 41.4)	
35 and older	735	429	36.9 (32.8, 41.2)	
**Maternal education**				
No formal education	925	806	46.6 (43.7, 49.4)	<0.0001
Primary	1,456	987	40.4 (37.2, 43.7)	
Secondary	1,152	401	25.8 (22.5, 29.4)	
Higher	162	16	9.0 (4.2, 18.3)	
**Cohabitation**				
Living together	2,652	1,540	36.8 (34.0, 39.6)	0.0666
Living separated	262	183	41.2 (36.0, 46.5)	
Never in union	520	282	35.1 (31.1, 39.4)	
Widowed, divorced, separated	261	204	43.8 (37.4, 50.5)	
**Age of sexual initiation, years**				
14 and under	936	605	39.3 (35.9, 42.7)	0.0209
15 to 16	1,372	891	39.4 (36.0, 42.8)	
17 and above	1,387	713	34.0 (30.7, 37.3)	
**Sexual autonomy**				
No/ not sure	1,033	752	42.1 (38.8, 45.5)	0.002
Yes	1,880	972	34.1 (30.8, 37.5)	
Father not present	781	486	38.3 (34.7, 42.1)	
**Safe-sex autonomy**				
No/ not sure	1,219	927	43.2 (40.3, 46.2)	<0.0001
Yes	1,695	797	32.0 (28.4, 35.8)	
Father not present	781	486	38.3 (34.7, 42.1)	
**Lifetime natality control**				
No	2,730	1,830	40.1 (37.9, 42.4)	<0.0001
Yes	965	380	28.2 (23.7, 33.3)	
**Antenatal care, visits**				
Less than four (< 4 visits)	801	585	42.2 (39.1, 45.4)	<0.0001
Four of more (≥ 4 visits)	1,680	712	29.8 (26.3, 33.5)	
Missing	1,215	912	42.9 (39.7, 46.2)	
**Newborn health visit**				
No	1,881	1,004	34.8 (32.1, 37.6)	<0.0001
Yes	595	296	33.2 (28.8, 37.9)	
Missing	1,219	910	42.7 (39.6, 46.0)	
**Breastfeeding**				
Currently	1,476	660	30.9 (28.1, 33.8)	<0.0001
Not currently	2,097	1,447	40.8 (38.3, 43.5)	
Never breastfed	122	103	45.7 (35.8, 55.9)	
**Healthcare decision**				
Husband, partner	748	451	37.7 (34.2, 41.2)	0.5470
Joint	1,581	891	36.0 (32.8, 39.4)	
Woman	585	381	39.5 (34.7, 44.4)	
Father not present	781	486	38.3 (34.7, 42.1)	
**Decision to visit family**				
Husband/ partner	375	229	38.0 (33.3, 42.9)	0.2777
Joint	1,747	968	35.6 (32.6, 38.9)	
Woman	792	527	40.0 (35.8, 44.3)	
Father not present	781	486	38.3 (34.7, 42.1)	
**Work outside of home**				
No	1,037	488	32.0 (27.8, 36.5)	0.003
Yes	2,659	1,722	39.3 (37.1, 41.6)	
**Paternal age-group, years**				
15 to 19	36	18	33.1 (21.0, 47.9)	0.1602
20 to 24	268	209	43.8 (37.8, 50.0)	
25 to 29	629	407	39.3 (34.6, 44.1)	
30 to 34	581	347	37.4 (32.2, 42.9)	
35 and older	1,336	711	34.7 (31.3, 38.3)	
Father not present	781	486	38.3 (34.7, 42.1)	
Missing	64	33	34.0 (26.0, 43.1)	
**Paternal education**				
No formal education	325	295	47.6 (43.1, 52.0)	<0.0001
Primary	865	683	44.1 (40.5, 47.8)	
Secondary	1,285	586	31.3 (27.4, 35.6)	
Higher	230	36	13.5 (8.5, 20.6)	
Father not present	781	486	38.3 (34.7, 42.1)	
Missing	209	124	37.3 (29.8, 45.4)	
**Source of water**				
Piped	1,003	346	25.7 (20.9, 31.1)	<0.0001
Fountain/ well	1,126	794	41.3 (37.9, 44.9)	
Spring/ surface/ rain	886	782	46.9 (43.9, 49.9)	
Other	643	277	30.1 (25.6, 35.1)	
Missing	38	11	22.1 (12.7, 35.5)	
**Sanitary system**				
Public sewage	246	128	34.2 (25.5, 44.1)	<0.0001
Septic tank	2,182	1,027	32.0 (29.2, 35.0)	
Open pit	197	203	50.8 (44.6, 56.9)	
No facility	1,010	824	44.9 (41.9, 48.0)	
Other	23	16	41.8 (25.6, 60.0)	
Missing	38	11	22.1 (12.7, 35.5)	
**Shared toilet**				
No	1,762	819	31.7 (28.6, 35.0)	<0.0001
Yes	885	556	38.6 (34.0, 43.3)	
No toilet	1,010	824	44.9 (41.9, 48.0)	
Missing	38	11	22.1 (12.7, 35.5)	
**Electricity in household**				
No	2,016	1,585	44.0 (41.7, 46.4)	<0.0001
Yes	1,642	614	27.2 (23.4, 31.4)	
Missing	38	11	22.1 (12.7, 35.5)	
**Refrigerator in household**				
No	2,169	1,743	44.6 (42.4, 46.8)	<0.0001
Yes	1,488	456	23.5 (19.4, 28.0)	
Missing	38	11	22.1 (12.7, 35.5)	
**Type of flooring**				
Earth, sand, gravel	1,659	1,384	45.5 (43.2, 47.8)	<0.0001
Cement	1,506	649	30.1 (26.4, 34.1)	
Ceramic, stone	465	126	21.3 (15.4, 28.7)	
Other	26	39	60.0 (37.8, 78.8)	
Missing	38	11	22.1 (12.7, 35.5)	
**Cooking fuel**				
Gas	1,958	812	29.3 (25.6, 33.3)	<0.0001
Charcoal	550	362	39.7 (36.3, 43.3)	
Biomass	1,049	945	47.4 (44.8, 50.0)	
Other	99	80	44.5 (34.6, 54.7)	
Missing	38	11	22.1 (12.7, 35.5)	
**Household size, members**				
1 to 4	937	560	37.4 (33.8, 41.1)	0.1019
5 to 6	1,220	813	40.0 (36.5, 43.6)	
7 and above	1,539	837	35.2 (32.2, 38.4)	
**Eligible women in household, count**				
One	2,755	1,736	38.7 (36.4, 41.0)	0.046
Two	646	343	34.7 (29.9, 39.9)	
Three or more	294	130	30.6 (24.4, 37.6)	
**Eligible children in household, count**				
One	954	411	30.1 (26.9, 33.5)	<0.0001
Two	1,588	1,049	39.8 (36.6, 43.0)	
Three or more	1,154	750	39.4 (36.4, 42.5)	
**Wealth Index**				
Poorest	676	615	47.6 (44.3, 51.0)	<0.0001
Poorer	752	618	45.1 (42.2, 48.1)	
Middle	812	504	38.3 (34.1, 42.7)	
Wealthier	800	307	27.7 (22.1, 34.1)	
Wealthiest	655	165	20.1 (15.3, 26.1)	
**Area of residence**				
Urban	2,425	1,129	31.8 (28.5, 35.2)	<0.0001
Rural	1,270	1,080	46.0 (43.5, 48.5)	
**Provinces**				
Bengo	49	33	39.8 (34.5, 45.3)	<0.0001
Benguela	364	177	32.7 (28.0, 37.7)	
Bié	149	149	50.0 (43.9, 56.1)	
Cabinda	98	28	22.4 (16.6, 29.4)	
Cuando-Cubango	52	43	45.2 (36.6, 54.1)	
Cuanza Norte	45	40	47.1 (39.8, 54.4)	
Cuanza Sul	241	226	48.3 (42.5, 54.2)	
Cunene	139	77	35.8 (30.8, 41.1)	
Huíla	314	234	42.7 (36.6, 49.0)	
Huambo	295	230	43.9 (35.6, 52.5)	
Luanda	1,197	520	30.3 (24.4, 36.9)	
Lunda Norte	102	64	38.5 (30.9, 46.8)	
Lunda Sul	63	49	43.9 (38.4, 49.6)	
Malanje	172	91	34.8 (28.9, 41.2)	
Moxico	75	48	39.0 (30.9, 47.7)	
Namibe	53	29	35.5 (30.1, 41.3)	
Uíge	201	143	41.6 (36.3, 47.1)	
Zaire	88	29	24.8 (19.6, 31.0)	
**Wealth Index**				
Poorest	676	615	47.6 (44.3, 51.0)	<0.0001
Poorer	752	618	45.1 (42.2, 48.1)	
Middle	812	504	38.3 (34.1, 42.7)	
Wealthier	800	307	27.7 (22.1, 34.1)	
Wealthiest	655	165	20.1 (15.3, 26.1)	
**Area of residence**				
Urban	2,425	1,129	31.8 (28.5, 35.2)	<0.0001
Rural	1,270	1,080	46.0 (43.5, 48.5)	
**Provinces**				
Bengo	49	33	39.8 (34.5, 45.3)	<0.0001
Benguela	364	177	32.7 (28.0, 37.7)	
Bié	149	149	50.0 (43.9, 56.1)	
Cabinda	98	28	22.4 (16.6, 29.4)	
Cuando-Cubango	52	43	45.2 (36.6, 54.1)	
Cuanza Norte	45	40	47.1 (39.8, 54.4)	
Cuanza Sul	241	226	48.3 (42.5, 54.2)	
Cunene	139	77	35.8 (30.8, 41.1)	
Huíla	314	234	42.7 (36.6, 49.0)	
Huambo	295	230	43.9 (35.6, 52.5)	
Luanda	1,197	520	30.3 (24.4, 36.9)	
Lunda Norte	102	64	38.5 (30.9, 46.8)	
Lunda Sul	63	49	43.9 (38.4, 49.6)	
Malanje	172	91	34.8 (28.9, 41.2)	
Moxico	75	48	39.0 (30.9, 47.7)	
Namibe	53	29	35.5 (30.1, 41.3)	
Uíge	201	143	41.6 (36.3, 47.1)	
Zaire	88	29	24.8 (19.6, 31.0)	

^1^ Values are weighted counts (n), and prevalence of stunting (95% confidence interval). P-values were obtained using the design-based F-score to account for the distinct weights of each observation resulting from the complex sampling procedure used.

### Outcome data

**Fig 2:**
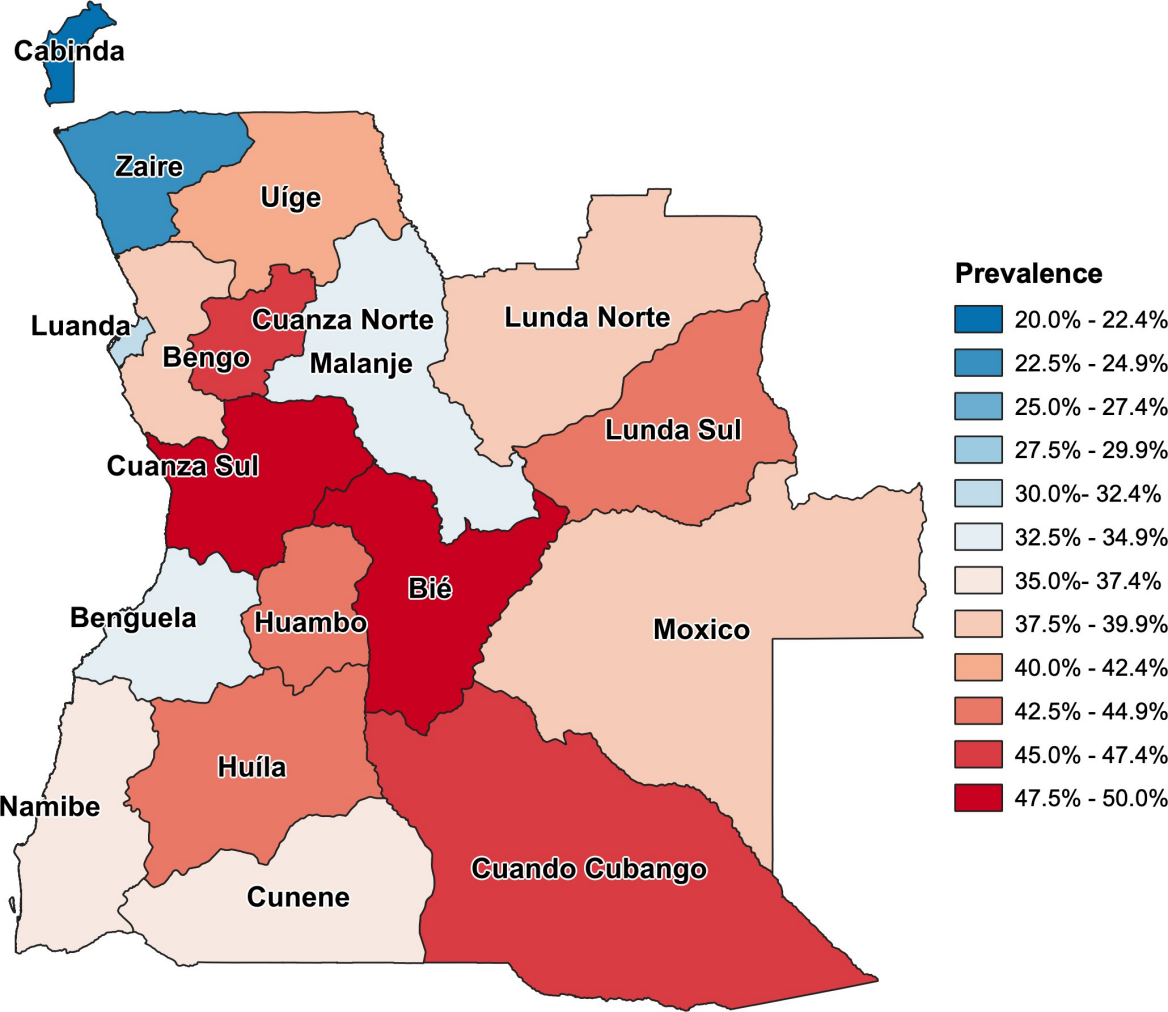
Map of the Republic of Angola displaying prevalence of stunting by province. Map shows the territory of the Republic of Angola with the 18 provinces color-coded according to prevalence of stunting among children 0 to 59 months of age estimated in the Angola 2015–2016 DHS (Table 1). Base layer map shapefile was obtained from GADM (https://gadm.org/maps/AGO.html) [40]. Terms and conditions of use available from https://gadm.org/license.html.

Prevalence of stunting was 37.4% (95% CI, 35.3% to 39.6%) among children 0 to 59 months of age in Angola, and varied according to characteristics of children, parents, and households (Table 1). Stunting was associated with sex of child (p<0.0001), child age (p<0.0001), birthweight (p<0.0001), and recent diarrhea (p<0.0001) (Table 1). In terms of maternal characteristics, stunting was associated with maternal education (p<0.0001), maternal employment (p = 0.003), mother’s age at sexual initiation (p = 0.021), autonomy to refuse sex (p = 0.002), autonomy to request condom (p<0.0001), lifetime use of natality control (p<0.0001), completion of minimal number of ANC visits (p<0.0001), newborn health visit (p<0.0001), and duration of breast feeding (p<0.0001) (Table 1). Paternal education (p<0,0001) was also associated with stunting (Table 1). Several characteristics of households were associated with stunting, including source of water (p<0.0001), sanitary system (p<0.0001), toilet-sharing (p<0.0001), electricity in household (p<0.0001), refrigerator ownership (p<0.0001), type of flooring (p<0.0001), and cooking fuel used (p<0.0001). In terms of household composition, number of women 15- to 49-year-old (p = 0.046) and children (p<0.001) in the household were associated with stunting (Table 1). Stunting was associated with quintiles of wealth index (p<0.0001), area of residence (p<0.0001), and province (p<0.0001) (Table 1) (Fig 2).

### Measure of effect

**Fig 3:**
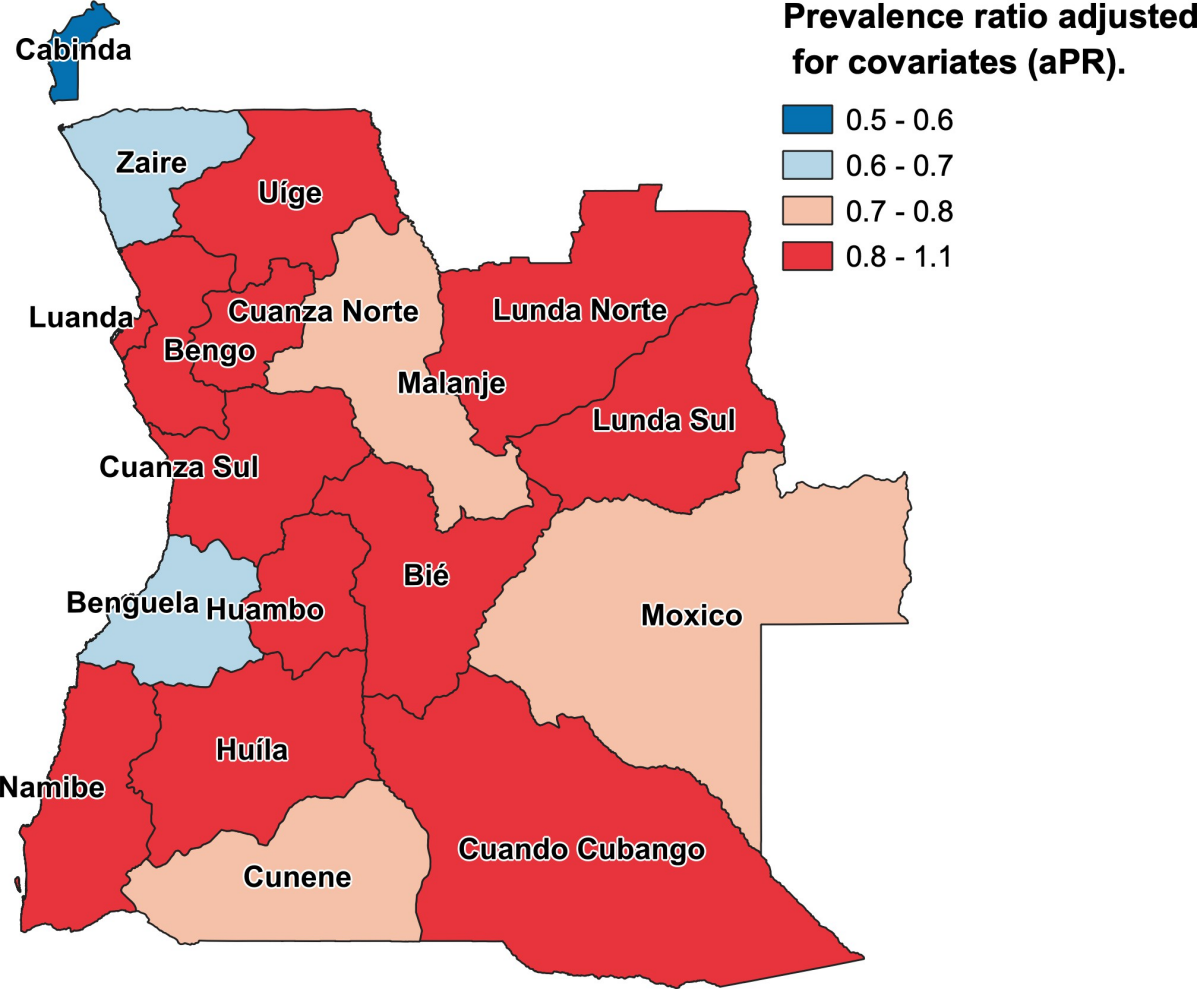
Map of the Republic of Angola displaying prevalence ratio of stunting for each province relative to Luanda. Map shows the territory of the Republic of Angola with the 18 provinces color-coded to display adjusted prevalence ratio of stunting among children 0 to 59 months of age relative to Luanda. Adjusted prevalence ratios were obtained using fixed-effects multivariable Poisson regression model 3 (Table 2). Base layer map shapefile was obtained from GADM (https://gadm.org/maps/AGO.html) [40]. Terms and conditions of use available from https://gadm.org/license.html.

Crude and adjusted prevalence ratios for each level of exposure variables are reported (Table 2). Crude and adjusted absolute risk differences are also presented (S4 Table). For most exposure variables, the adjusted prevalence ratios (aPRs) estimated with partially adjusted models (model 1 and model 2) were similar to those obtained with the fully adjusted model (model 3) (Table 2). Prevalence ratios estimated with fully adjusted model (model 3) are described here. Risk of stunting was higher among boys, relative to girls: aPR = 1.21 (95% CI, 1.12 to 1.30; p< 0.001) (Table 2). Risk of stunting increased sharply in the second, aPR = 2.14 (95% CI, 1.82 to 2.52; p< 0.001), third, 2.46 (95% CI, 1.99 to 3.04; p< 0.001), and fourth years of life, aPR = 2.18 (95% CI, 1.75 to 2.71; p< 0.001) (Table 2). Risk of stunting increased with birth order: third and fourth-born, aPR = 1.24 (95% CI, 1.05 to 1.47; p = 0.011), and fifth or above, aPR = 1.36 (95% CI, 1.11 to 1.68; p = 0.004) (Table 2). Children born with low birthweight had higher risk of stunting, aPR = 1.35 (95% CI, 1.13 to 1.60; p = 0.001); while those with high birthweight had a lower risk: aPR = 0.75 (95% CI, 0.63 to 0.89; p = 0.001) (Table 2). Risk of stunting was higher among children with recent diarrhea: aPR = 1.24 (95% CI, 1.11 to 1.37; p< 0.001) (Table 2). Children of young mothers experienced higher risk of stunting: 15- to 19-year-old, aPR = 1.38 (95% CI, 1.08 to 1.75; p = 0.009), and 20- to 24-year-old, aPR = 1.28 (95% CI, 1.06 to 1.53; p = 0.009) (Table 2). Risk of stunting decreased when mothers reached secondary education, aPR = 0.80 (95% CI, 0.67 to 0.95; p = 0.011), or higher education, aPR = 0.36 (95% CI, 0.19 to 0.68; p = 0.002) (Table 2). Risk of stunting did not change according to other maternal characteristics or child-care behaviors, after adjusting to confounding variables (Table 2). Risk of stunting was higher among children of fathers 20- to 24-year-old, aPR = 1.28 (95% CI, 1.03 to 1.58; p = 0.024), and decreased when fathers reached higher education, aPR = 0.61 (95% CI, 0.39 to 0.97; p = 0.036) (Table 2). Consuming water from fountains or wells, or open sources of water (springs, surface, or rain), increased risk for stunting: aPR = 1.30 (95% CI, 1.07 to 1.57; p = 0.008), aPR = 1.30 (95% CI, 1.06 to 1.60; p = 0.012), respectively (Table 2). Children residing in households using open pit for disposal of fecal matter had a higher risk of stunting: aPR = 1.22 (95% CI, 1.06 to 1.40; p = 0.005). Unexpectedly, lack of access to sanitary facility was not associated with increased risk of stunting, aPR = 1.02 (95% CI, 0.89 to 1.17; p = 0.738) (Table 2). Risk of stunting was not associated with presence of electricity in the household after adjusting for refrigerator ownership, but children residing in households without refrigerator had higher risk of stunting: aPR = 1.47 (95% CI, 1.19 to 1.83; p< 0.001). Household flooring and cooking fuel did not influence the risk of stunting (Table 2). In terms of household composition, total number of members and women of reproductive age did not influence risk of stunting, while risk of stunting was lower in households with a single child: aPR = 0.88 (95% CI, 0.78 to 0.99; p = 0.035) (Table 2). Wealth index and area of residence (rural or urban) were not associated with stunting, after adjusting for confounding variables (Table 2). However, risk of stunting was lower in certain provinces, even after accounting for all individual and household-level variables described previously (Fig 3; Table 2).

**Table 2:** Crude and adjusted prevalence ratio of stunting according to characteristics of participants and households.

Characteristic	Crude		Model 1		Model 2		Model 3	
	PR (95% CI)	p-value	aPR (95% CI)	p-value	aPR (95% CI)	p-value	aPR (95% CI)	p-value
**Sex of child**								
Female	1.0	-	1.0	-	1.0	-	1.0	-
Male	1.23 (1.13,1.33)	<0.001	1.21 (1.12, 1.30)	<0.001	1.21 (1.12, 1.30)	<0.001	1.21 (1.12, 1.30)	<0.001
**Child age, months**								
0 to 11	1.0	-	1.0	-	1.0	-	1.0	-
12 to 23	2.20 (1.87, 2.60)	<0.001	2.17 (1.86, 2.54)	<0.001	2.17 (1.84, 2.56)	<0.001	2.14 (1.82, 2.53)	<0.001
24 to 35	2.35 (2.00, 2.76)	<0.001	2.47 (2.10, 2.91)	<0.001	2.48 (2.00, 3.07)	<0.001	2.46 (1.99, 3.04)	<0.001
36 to 47	2.02 (1.71, 2.39)	<0.001	2.14 (1.81, 2.54)	<0.001	2.15 (1.73, 2.67)	<0.001	2.18 (1.75, 2.71)	<0.001
48 to 59	1.59 (1.33, 1.91)	<0.001	1.65 (1.38, 1.99)	<0.001	1.68 (1.33, 2.12)	<0.001	1.69 (1.34, 2.15)	<0.001
**Birth order**								
First	1.0	-	1.0	-	1.0	-	1.0	-
Second	1.01 (0.88, 1.16)	0.898	1.02 (0.88, 1.18)	0.796	1.02 (0.88, 1.19)	0.775	1.04 (0.89, 1.20)	0.641
Third and fourth	1.13 (0.97, 1.31)	0.107	1.23 (1.05, 1.44)	0.011	1.24 (1.04, 1.47)	0.014	1.24 (1.05, 1.47)	0.011
Fifth and above	1.15 (1.01, 1.30)	0.036	1.33 (1.10, 1.62)	0.004	1.36 (1.10, 1.67)	0.004	1.36 (1.11, 1.68)	0.004
**Birthweight (grams)**								
Low (< 2,500)	1.48 (1.22, 1.80)	<0.001	-	-	-	-	1.35 (1.13, 1.60)	0.001
Normal (2,500 to 3,999)	1.0	-	-	-	-	-	1.0	-
High (≥ 4,000)	0.86 (0.70, 1.06)	0.151	-	-	-	-	0.75 (0.63, 0.89)	0.001
Not weighed at birth	1.57 (1.37, 1.79)	<0.001	-	-	-	-	1.08 (0.97, 1.20)	0.153
Missing	1.47 (1.17, 1.85)	0.001	-	-	-	-	1.13 (0.94, 1.36)	0.191
**Diarrhea in last 2 weeks**								
No	1.0	-	-	-	-	-	1.0	-
Yes	1.28 (1.15, 1.43)	<0.001	-	-	-	-	1.24 (1.11, 1.37)	<0.001
Missing	0.54 (0.22, 1.28)	0.158	-	-	-	-	0.25 (0.09, 0.73)	0.011
**Fever in last 2 weeks**								
No	1.0	-	-	-	-	-	1.0	-
Yes	1.09 (0.96, 1.23)	0.179	-	-	-		0.99 (0.88, 1.11)	0.805
Missing	0.96 (0.52, 1.79)	0.908	-	-	-	-	3.14 (0.69, 14.1)	0.137
**Cough in last 2 weeks**								
No	1.0	-	-	-	-	-	1.0	-
Yes	1.00 (0.87, 1.15)	0.993	-	-	-		0.98 (0.87, 1.10)	0.694
Missing	1.00 (0.45, 2.22)	0.991	-	-	-	-	0.75 (0.15, 3.71)	0.720
**Maternal age, years**								
15 to 19	1.00 (0.84, 1.19)	0.975	1.32 (1.06, 1.65)	0.013	1.41 (1.11, 1.78)	0.005	1.38 (1.08, 1.75)	0.009
20 to 24	1.11 (0.98, 1.26)	0.098	1.28 (1.07, 1.53)	0.008	1.32 (1.10, 1.59)	0.003	1.28 (1.06, 1.53)	0.009
25 to 29	0.95 (0.82, 1.11)	0.526	1.08 (0.92, 1.27)	0.313	1.12 (0.95, 1.31)	0.174	1.10 (0.94, 1.29)	0.240
30 to 34	0.98 (0.82, 1.16)	0.782	1.07 (0.94, 1.23)	0.306	1.08 (0.94, 1.24)	0.257	1.09 (0.95, 1.26)	0.215
35 and older	1.0	-	1.0	-	1.0	-	1.0	-
**Maternal education,**								
No formal education	1.0	-	1.0	-	1.0	-	1.0	-
Primary	0.87 (0.79, 0.95)	0.003	0.96 (0.87, 1.06)	0.430	0.97 (0.87, 1.07)	0.506	0.98 (0.89, 1.09)	0.746
Secondary	0.55 (0.48, 0.64)	<0.001	0.77 (0.66, 0.91)	0.003	0.78 (0.66, 0.92)	0.004	0.80 (0.67, 0.95)	0.011
Higher	0.19 (0.09, 0.41)	<0.001	0.36 (0.19, 0.68)	0.002	0.35 (0.19, 0.64)	0.001	0.36 (0.19, 0.68)	0.002
**Cohabitation status**								
Living together	1.0	-	1.0	-	1.0	-	1.0	-
Living separated	1.12 (0.97, 1.29)	0.124	1.05 (0.93, 1.20)	0.423	1.02 (0.90, 1.17)	0.731	1.02 (0.90, 1.17)	0.748
Widowed, divorced	1.19 (1.01, 1.40)	0.033	1.08 (0.91, 1.30)	0.373	1.13 (0.93, 1.38)	0.223	1.12 (0.92, 1.36)	0.275
Never in a union	0.96 (0.83, 1.11)	0.549	1.09 (0.91, 1.29)	0.356	1.13 (0.93, 1.38)	0.215	1.12 (0.92, 1.37)	0.247
**Sexual initiation, years**								
14 and younger	1.16 (1.02, 1.31)	0.024	-	-	0.92 (0.82, 1.04)	0.185	0.93 (0.83, 1.05)	0.250
15 to 16	1.16 (1.03, 1.30)	0.011	-	-	0.98 (0.87, 1.09)	0.660	0.98 (0.87, 1.09)	0.652
17 and older	1.0	-	-	-	1.0	-	1.0	-
**Sexual autonomy**								
Yes	1.0	-	-	-	1.0	-	1.0	-
No	1.24 (1.10, 1.39)	0.001	-	-	0.99 (0.88, 1.11)	0.841	0.99 (0.88, 1.12)	0.867
**Safe sex autonomy**			-					
Yes	1.0	-	-	-	1.0	-	1.0	-
No	1.35 (1.19, 1.54)	<0.001	-	-	1.02 (0.90, 1.16)	0.753	1.01 (0.89, 1.15)	0.852
**Lifetime natality control**								
Yes	1.0	-	-	-	1.0	-	1.0	-
No	1.42 (1.20, 1.69)	<0.001	-	-	1.04 (0.89, 1.20)	0.644	1.05 (0.90, 1.22)	0.545
**Antenatal care, visits**								
< 4 visits	1.42 (1.24, 1.63)	<0.001	-	-	1.12 (0.99, 1.27)	0.066	1.10 (0.97, 1.24)	0.124
≥ 4 visits	1.0	-	-	-	1.0	-	1.0	-
Missing	1.44 (1.26, 1.65)	<0.001	-	-	1.27 (0.83, 1.97)	0.273	1.22 (0.78, 1.90)	0.390
**Newborn health visit**								
No	1.0	-	-	-	1.0	-	1.0	-
Yes	0.95 (0.83, 1.10)	0.504	-	-	1.05 (0.92, 1.20)	0.471	1.01 (0.88, 1.15)	0.918
Missing	1.23 (1.11, 1.35)	<0.001	-	-	0.81 (0.53, 1.25)	0.341	0.85 (0.55, 1.32)	0.464
**Breastfeeding**								
Currently	1.0	-	-	-	1.0	-	1.0	-
Not currently	1.32 (1.20, 1.46)	<0.001	-	-	1.02 (0.90, 1.16)	0.775	1.03 (0.90, 1.17)	0.698
Never breastfed	1.48 (1.18, 1.85)	0.001	-	-	1.10 (0.88, 1.36)	0.413	1.09 (0.87, 1.36)	0.466
**Healthcare decision**								
Joint	1.0	-	-	-	1.0	-	1.0	-
Husband/ partner/ other	1.04 (0.93, 1.18)	0.470	-	-	0.99 (0.88, 1.11)	0.866	0.99 (0.88, 1.11)	0.846
Woman	1.09 (0.97, 1.24)	0.158	-	-	1.12 (0.98, 1.29)	0.088	1.12 (0.98, 1.28)	0.105
**Decision to visit family**								
Joint	1.0	-	-	-	1.0	-	1.0	-
Husband/ partner/ other	1.07 (0.92, 1.24)	0.411	-	-	1.00 (0.85, 1.17)	0.990	0.98 (0.85, 1.15)	0.845
Woman	1.12 (1.00, 1.26)	0.055	-	-	1.07 (0.94, 1.21)	0.311	1.05 (0.93, 1.19)	0.418
**Work outside of home**								
Not working	0.81 (0.71, 0.94)	0.004	-	-	0.94 (0.82, 1.07)	0.348	0.94 (0.82, 1.08)	0.377
Working	1.0	-	-	-	1.0	-	1.0	-
**Paternal age, years**								
15 to 19	0.95 (0.62, 1.45)	0.821	1.05 (0.67, 1.65)	0.834	1.05 (0.66, 1.66)	0.835	1.04 (0.66, 1.66)	0.853
20 to 24	1.26 (1.08, 1.47)	0.003	1.25 (1.02, 1.53)	0.033	1.28 (1.03, 1.58)	0.023	1.28 (1.03, 1.58)	0.024
25 to 29	1.13 (0.98, 1.30)	0.090	1.10 (0.93, 1.29)	0.281	1.10 (0.93, 1.31)	0.270	1.11 (0.93, 1.32)	0.251
30 to 34	1.08 (0.90, 1.28)	0.410	1.07 (0.90, 1.26)	0.441	1.07 (0.90, 1.27)	0.428	1.09 (0.92, 1.28)	0.327
35 and older	1.0	-	1.0	-	1.0	-	1.0	-
Missing	0.98 (0.75, 1.28)	0.876	0.87 (0.68, 1.11)	0.258	0.86 (0.68, 1.10)	0.233	0.86 (0.68, 1.10)	0.221
**Paternal education,**								
No formal education	1.0	-	1.0	-	1.0	-	1.0	-
Primary	0.93 (0.82, 1.05)	0.235	0.99 (0.87, 1.18)	0.831	0.98 (0.86, 1.11)	0.761	0.97 (0.85, 1.10)	0.593
Secondary	0.66 (0.56, 0.77)	<0.001	0.91 (0.77, 1.06)	0.225	0.90 (0.77, 1.05)	0.178	0.89 (0.76, 1.04)	0.143
Higher	0.28 (0.18, 0.45)	<0.001	0.64 (0.40, 1.01)	0.053	0.63 (0.40, 1.00)	0.048	0.61 (0.39, 0.97)	0.036
Missing	0.78 (0.63, 0.98)	0.031	0.91 (0.75, 1.10)	0.341	0.89 (0.74, 1.08)	0.242	0.90 (0.74, 1.09)	0.281
**Source of water**								
Piped	1.0	-	1.0	-	1.0	-	1.0	-
Fountain, well	1.61 (1.30, 2.00)	<0.001	1.30 (1.07, 1.58)	0.008	1.30 (1.07, 1.57)	0.008	1.30 (1.07, 1.57)	0.008
Spring/ surface/ rain	1.83 (1.48, 2.26)	<0.001	1.31 (1.07, 1.61)	0.010	1.30 (1.06, 1.60)	0.011	1.30 (1.06, 1.60)	0.012
Other	1.17 (0.92, 1.50)	0.204	1.09 (0.89, 1.33)	0.419	1.08 (0.88, 1.32)	0.461	1.09 (0.89, 1.34)	0.410
**Sanitary system**								
Septic tank	1.0	-	1.0	-	1.0	-	1.0	-
Public sanitary sewer	1.07 (0.82, 1.40)	0.625	1.15 (0.93, 1.42)	0.185	1.17 (0.95, 1.44)	0.140	1.21 (0.98, 1.51)	0.076
Open pit	1.59 (1.37, 1.83)	<0.001	1.23 (1.08, 1.41)	0.003	1.23 (1.07, 1.40)	0.003	1.22 (1.06, 1.40)	0.005
No sanitary facility	1.40 (1.26, 1.56)	<0.001	1.04 (0.90, 1.19)	0.617	1.03 (0.90, 1.18)	0.678	1.02 (0.89, 1.17)	0.738
Other	1.31 (0.85, 2.00)	0.220	1.21 (0.88, 1.68)	0.245	1.15 (0.83, 1.61)	0.403	1.15 (0.82, 1.61)	0.407
**Shared toilet**								
No	1.0	-	1.0	-	1.0	-	1.0	-
Yes	1.22 (1.05, 1.41)	0.011	1.12 (0.99, 1.26)	0.076	1.12 (0.99, 1.26)	0.069	1.11 (0.98, 1.25)	0.090
**Electricity**								
Yes	1.0	-	1.0	-	1.0	-	1.0	-
No	1.62 (1.39, 1.88)	<0.001	1.05 (0.89, 1.24)	0.551	1.05 (0.89, 1.24)	0.552	1.03 (0.87, 1.21)	0.730
**Refrigerator**								
Yes	1.0	-	1.0	-	1.0	-	1.0	-
No	1.90 (1.58, 2.28)	<0.001	1.48 (1.19, 1.84)	0.001	1.46 (1.18, 1.80)	<0.001	1.47 (1.19, 1.83)	<0.001
**Type of flooring**								
Earth/ sand/ gravel	1.51 (1.31, 1.74)	<0.001	1.05 (0.88, 1.26)	0.556	1.04 (0.87, 1.24)	0.672	1.03 (0.87, 1.23)	0.725
Cement	1.0	-	1.0	-	1.0	-	1.0	-
Ceramic/ stone	0.71 (0.52, 0.96)	0.024	1.03 (0.77, 1.36)	0.859	1.03 (0.78, 1.36)	0.854	1.08 (0.81, 1.45)	0.585
Other	1.99 (1.39, 2.85)	<0.001	1.73 (1.23, 2.42)	0.002	1.72 (1.23, 2.42)	0.002	1.75 (1.22, 2.52)	0.002
**Cooking fuel**								
Gas	1.0	-	1.0	-	1.0	-	1.0	-
Charcoal	1.35 (1.15, 1.59)	<0.001	0.94 (0.80, 1.11)	0.495	0.93 (0.79, 1.09)	0.384	0.90 (0.77, 1.06)	0.200
Biomass	1.62 (1.40, 1.87)	<0.001	1.02 (0.86, 1.21)	0.792	1.01 (0.85, 1.19)	0.929	0.98 (0.83, 1.15)	0.800
Other	1.52 (1.21, 1.90)	<0.001	1.31 (1.05, 1.63)	0.017	1.28 (1.04, 1.59)	0.022	1.24 (0.99, 1.57)	0.061
**Household size, residents**								
One to four	1.0	-	1.0	-	1.0	-	1.0	-
Five to six	1.07 (0.94, 1.22)	0.317	1.00 (0.86, 1.21)	0.951	1.00 (0.89, 1.13)	0.961	1.00 (0.89, 1.13)	0.982
Seven or more	0.94 (0.83, 1.07)	0.346	0.92 (0.79, 1.08)	0.307	0.92 (0.79, 1.08)	0.322	0.93 (0.80, 1.09)	0.393
**Paternal age, years**								
15 to 19	0.95 (0.62, 1.45)	0.821	1.05 (0.67, 1.65)	0.834	1.05 (0.66, 1.66)	0.835	1.04 (0.66, 1.66)	0.853
20 to 24	1.26 (1.08, 1.47)	0.003	1.25 (1.02, 1.53)	0.033	1.28 (1.03, 1.58)	0.023	1.28 (1.03, 1.58)	0.024
25 to 29	1.13 (0.98, 1.30)	0.090	1.10 (0.93, 1.29)	0.281	1.10 (0.93, 1.31)	0.270	1.11 (0.93, 1.32)	0.251
30 to 34	1.08 (0.90, 1.28)	0.410	1.07 (0.90, 1.26)	0.441	1.07 (0.90, 1.27)	0.428	1.09 (0.92, 1.28)	0.327
35 and older	1.0	-	1.0	-	1.0	-	1.0	-
Missing	0.98 (0.75, 1.28)	0.876	0.87 (0.68, 1.11)	0.258	0.86 (0.68, 1.10)	0.233	0.86 (0.68, 1.10)	0.221
**Paternal education,**								
No formal education	1.0	-	1.0	-	1.0	-	1.0	-
Primary	0.93 (0.82, 1.05)	0.235	0.99 (0.87, 1.18)	0.831	0.98 (0.86, 1.11)	0.761	0.97 (0.85, 1.10)	0.593
Secondary	0.66 (0.56, 0.77)	<0.001	0.91 (0.77, 1.06)	0.225	0.90 (0.77, 1.05)	0.178	0.89 (0.76, 1.04)	0.143
Higher	0.28 (0.18, 0.45)	<0.001	0.64 (0.40, 1.01)	0.053	0.63 (0.40, 1.00)	0.048	0.61 (0.39, 0.97)	0.036
Missing	0.78 (0.63, 0.98)	0.031	0.91 (0.75, 1.10)	0.341	0.89 (0.74, 1.08)	0.242	0.90 (0.74, 1.09)	0.281
**Source of water**								
Piped	1.0	-	1.0	-	1.0	-	1.0	-
Fountain, well	1.61 (1.30, 2.00)	<0.001	1.30 (1.07, 1.58)	0.008	1.30 (1.07, 1.57)	0.008	1.30 (1.07, 1.57)	0.008
Spring/ surface/ rain	1.83 (1.48, 2.26)	<0.001	1.31 (1.07, 1.61)	0.010	1.30 (1.06, 1.60)	0.011	1.30 (1.06, 1.60)	0.012
Other	1.17 (0.92, 1.50)	0.204	1.09 (0.89, 1.33)	0.419	1.08 (0.88, 1.32)	0.461	1.09 (0.89, 1.34)	0.410
**Sanitary system**								
Septic tank	1.0	-	1.0	-	1.0	-	1.0	-
Public sanitary sewer	1.07 (0.82, 1.40)	0.625	1.15 (0.93, 1.42)	0.185	1.17 (0.95, 1.44)	0.140	1.21 (0.98, 1.51)	0.076
Open pit	1.59 (1.37, 1.83)	<0.001	1.23 (1.08, 1.41)	0.003	1.23 (1.07, 1.40)	0.003	1.22 (1.06, 1.40)	0.005
No sanitary facility	1.40 (1.26, 1.56)	<0.001	1.04 (0.90, 1.19)	0.617	1.03 (0.90, 1.18)	0.678	1.02 (0.89, 1.17)	0.738
Other	1.31 (0.85, 2.00)	0.220	1.21 (0.88, 1.68)	0.245	1.15 (0.83, 1.61)	0.403	1.15 (0.82, 1.61)	0.407
**Shared toilet**								
No	1.0	-	1.0	-	1.0	-	1.0	-
Yes	1.22 (1.05, 1.41)	0.011	1.12 (0.99, 1.26)	0.076	1.12 (0.99, 1.26)	0.069	1.11 (0.98, 1.25)	0.090
**Electricity**								
Yes	1.0	-	1.0	-	1.0	-	1.0	-
No	1.62 (1.39, 1.88)	<0.001	1.05 (0.89, 1.24)	0.551	1.05 (0.89, 1.24)	0.552	1.03 (0.87, 1.21)	0.730
**Refrigerator**								
Yes	1.0	-	1.0	-	1.0	-	1.0	-
No	1.90 (1.58, 2.28)	<0.001	1.48 (1.19, 1.84)	0.001	1.46 (1.18, 1.80)	<0.001	1.47 (1.19, 1.83)	<0.001
**Type of flooring**								
Earth/ sand/ gravel	1.51 (1.31, 1.74)	<0.001	1.05 (0.88, 1.26)	0.556	1.04 (0.87, 1.24)	0.672	1.03 (0.87, 1.23)	0.725
Cement	1.0	-	1.0	-	1.0	-	1.0	-
Ceramic/ stone	0.71 (0.52, 0.96)	0.024	1.03 (0.77, 1.36)	0.859	1.03 (0.78, 1.36)	0.854	1.08 (0.81, 1.45)	0.585
Other	1.99 (1.39, 2.85)	<0.001	1.73 (1.23, 2.42)	0.002	1.72 (1.23, 2.42)	0.002	1.75 (1.22, 2.52)	0.002
**Cooking fuel**								
Gas	1.0	-	1.0	-	1.0	-	1.0	-
Charcoal	1.35 (1.15, 1.59)	<0.001	0.94 (0.80, 1.11)	0.495	0.93 (0.79, 1.09)	0.384	0.90 (0.77, 1.06)	0.200
Biomass	1.62 (1.40, 1.87)	<0.001	1.02 (0.86, 1.21)	0.792	1.01 (0.85, 1.19)	0.929	0.98 (0.83, 1.15)	0.800
Other	1.52 (1.21, 1.90)	<0.001	1.31 (1.05, 1.63)	0.017	1.28 (1.04, 1.59)	0.022	1.24 (0.99, 1.57)	0.061
**Household size, residents**								
One to four	1.0	-	1.0	-	1.0	-	1.0	-
Five to six	1.07 (0.94, 1.22)	0.317	1.00 (0.86, 1.21)	0.951	1.00 (0.89, 1.13)	0.961	1.00 (0.89, 1.13)	0.982
Seven or more	0.94 (0.83, 1.07)	0.346	0.92 (0.79, 1.08)	0.307	0.92 (0.79, 1.08)	0.322	0.93 (0.80, 1.09)	0.393
**Eligible women, number**								
One	1.0	-	1.0	-	1.0	-	1.0	-
Two	0.90 (0.78, 1.04)	0.140	1.07 (0.92, 1.24)	0.382	1.06 (0.92, 1.23)	0.426	1.05 (0.91, 1.22)	0.473
Three or more	0.79 (0.63, 0.99)	0.042	1.10 (0.88, 1.39)	0.399	1.10 (0.88, 1.39)	0.403	1.08 (0.86, 1.35)	0.520
**Eligible children, number**								
One	0.76 (0.67, 0.85)	<0.001	0.87 (0.78, 0.97)	0.011	0.86 (0.76, 0.97)	0.015	0.88 (0.78, 0.99)	0.035
Two	1.0	-	1.0	-	1.0	-	1.0	-
Three or more	0.99 (0.89, 1.10)	0.850	0.96 (0.88, 1.06)	0.453	0.97 (0.88, 1.06)	0.510	0.96 (0.88, 1.06)	0.424
**Wealth Index**								
Poorest	1.24 (1.09, 1.42)	0.001	0.95 (0.77, 1.19)	0.675	0.97 (0.78, 1.20)	0.751	0.99 (0.80, 1.23)	0.930
Poorer	1.18 (1.04, 1.34)	0.012	0.91 (0.75, 1.09)	0.301	0.92 (0.77, 1.10)	0.377	0.94 (0.79, 1.12)	0.486
Middle	1.0	-	1.0	-	1.0	-	1.0	-
Wealthier	0.72 (0.57, 0.92)	0.007	1.05 (0.85, 1.31)	0.643	1.05 (0.84, 1.31)	0.660	1.03 (0.83, 1.29)	0.766
Wealthiest	0.53 (0.40, 0.69)	<0.001	1.09 (0.75, 1.58)	0.652	1.11 (0.77, 1.60)	0.579	1.08 (0.75, 1.56)	0.682
**Area of residence**								
Urban	1.0	-	1.0	-	1.0	-	1.0	-
Rural	1.45 (1.29, 1.63)	<0.001	1.01 (0.89, 1.15)	0.892	1.01 (0.89, 1.14)	0.890	0.98 (0.87, 1.11)	0.748
**Provinces**								
Bengo	1.31 (1.03, 1.68)	0.030	0.90 (0.71, 1.13)	0.356	0.86 (0.68, 1.09)	0.211	0.91 (0.71, 1.16)	0.426
Benguela	1.08 (0.84, 1.39)	0.558	0.73 (0.58, 0.91)	0.005	0.70 (0.56, 0.87)	0.002	0.70 (0.56, 0.88)	0.002
Bié	1.65 (1.30, 2.10)	<0.001	0.90 (0.72, 1.12)	0.333	0.89 (0.71, 1.10)	0.272	0.91 (0.73, 1.14)	0.398
Cabinda	0.74 (0.52, 1.05)	0.093	0.55 (0.41, 0.73)	<0.001	0.56 (0.42, 0.74)	<0.001	0.57 (0.43, 0.76)	<0.001
Cuando-Cubango	1.49 (1.12, 1.98)	0.006	0.92 (0.70, 1.20)	0.529	0.92 (0.70, 1.20)	0.537	0.92 (0.70, 1.21)	0.541
Cuanza Norte	1.55 (1.20, 2.01)	0.001	0.99 (0.78, 1.25)	0.917	0.98 (0.78, 1.24)	0.876	0.98 (0.77, 1.24)	0.871
Cuanza Sul	1.60 (1.26, 2.03)	<0.001	0.88 (0.71, 1.10)	0.265	0.86 (0.69, 1.06)	0.165	0.88 (0.70, 1.08)	0.201
Cunene	1.18 (0.92, 1.52)	0.191	0.75 (0.59, 0.95)	0.018	0.75 (0.59, 0.96)	0.020	0.78 (0.61, 0.99)	0.049
Huíla	1.41 (1.10, 1.82)	0.008	0.82 (0.65, 1.03)	0.094	0.81 (0.64, 1.02)	0.073	0.82 (0.65, 1.04)	0.099
Huambo	1.45 (1.09, 1.92)	0.010	0.84 (0.66, 1.09)	0.189	0.84 (0.66, 1.07)	0.160	0.85 (0.67, 1.09)	0.205
Luanda	1.0	-	1.0	-	1.0	-	1.0	-
Lunda Norte	1.27 (0.95, 1.71)	0.107	0.84 (0.65, 1.10)	0.198	0.84 (0.64, 1.09)	0.186	0.88 (0.67, 1.16)	0.355
Lunda Sul	1.45 (1.14, 1.85)	0.003	1.01 (0.81, 1.24)	0.958	1.00 (0.80, 1.25)	0.990	1.07 (0.85, 1.34)	0.556
Malanje	1.15 (0.87, 1.51)	0.317	0.73 (0.56, 0.94)	0.015	0.72 (0.55, 0.93)	0.011	0.72 (0.56, 0.93)	0.013
Moxico	1.29 (0.95, 1.74)	0.098	0.74 (0.57, 0.98)	0.034	0.75 (0.57, 0.98)	0.036	0.76 (0.58, 0.99)	0.049
Namibe	1.17 (0.90, 1.52)	0.229	0.86 (0.67, 1.10)	0.221	0.84 (0.66, 1.08)	0.168	0.86 (0.68, 1.10)	0.230
Uíge	1.37 (1.08, 1.76)	0.011	0.86 (0.68, 1.09)	0.208	0.84 (0.67, 1.08)	0.155	0.87 (0.69, 1.10)	0.258
Zaire	0.82 (0.60, 1.12)	0.210	0.57 (0.42, 0.77)	<0.001	0.60 (0.44, 0.81)	0.001	0.63 (0.47, 0.84)	0.002

Numbers reported are crude prevalence ratio (PR) and prevalence ratio adjusted for confounding using multivariable, fixed-effects, Poisson regression (aPR), 95% confidence interval (95% CI) of prevalence ratio, and p-value of the hypothesis test that prevalence of each level of exposure equals the prevalence for the baseline level of variable.

### Stratified analysis

Effect modification by child age could be detected for child sex, birth order, birthweight, maternal age, paternal age, water source, sanitary system, and refrigerator ownership (Table 3). Effect of child sex was stronger among children who were 0 to 5 months and 6 to 23 months of age. Comparing to girls, boys were at greater risk for stunting during the first two years of life, than after the second year (Table 3). In the 0 to 5 months age-group, risk of stunting was higher among first-born children, but in the 24 months of age and older age-group, first-born children were at lower risk of stunting (Table 3). Low birthweight increased risk of stunting, but the effect size was larger in the first six months of life (Table 3). The effect of maternal age on risk of stunting was only detectable in children 24 months of age and older. Completion of antenatal care or newborn health visit did not show any association with stunting at any age-group. Breast feeding was associated with risk of stunting in children 6 to 23 months of age, but not in those 0 to 5 months and 24 months of age and older, suggesting the presence of effect modification that could not be fully detected with the sample available (Table 3). Paternal age seemed to have a strong effect for children in the 0 to 5 and 6 to 23 months of age groups, but not among older children (Table 3). Association of recent episode of diarrhea with stunting was similar for children 6 to 23 and 24 months of age and older, but not detected among children 0 to 5 months of age, indicating possible effect modification. Source of water had a stronger effect among children 0 to 5 months of age, while sanitary system had a greater effect in children 24 months of age and older (Table 3). Lack of refrigerator increased the risk of stunting, and stratified analysis showed a gradient of effect according to age-group; effect sizes were larger among younger children (Table 3). Effect modification by age-group was investigated for all variables using model 3, but only variables relevant to stratified analysis are presented on Table 3. Exposures that were not associated with stunting in the multivariable analysis model 3 (Table 2), not associated with stunting in the stratified analysis, and not expected to interact with child age-group were not presented on Table 3.

**Table 3:** Prevalence ratio adjusted for covariates (aPR), according to characteristics of participants and households stratified on child age-group.

Characteristic	Child age-group								
	All ages		0 to 5 months		6 to 23 months			> 24 months	
	aPR (95% CI)	p-value	aPR (95% CI)	p-value	aPR (95% CI)	p-value		aPR (95% CI)	p-value
**Sex of child**									
Female	1.0	-	1.0	-	1.0	-		1.0	-
Male	1.20 (1.12, 1.30)	<0.001	1.34 (0.92, 1.95)	0.125	1.34 (1.16, 1.56)	<0.001		1.14 (1.05, 1.25)	0.003
**Birth order**									
First	1.0	-	1.0	-	1.0	-		1.0	-
Second	1.02 (0.88, 1.20)	0.754	0.36 (0.17, 0.75)	0.007	0.83 (0.64, 1.07)	0.145		1.20 (1.00, 1.43)	0.052
Third and fourth	1.24 (1.05, 1.46)	0.013	0.39 (0.19, 0.84)	0.015	1.13 (0.86, 1.48)	0.369		1.44 (1.16, 1.79)	0.001
Fifth and above	1.34 (1.09, 1.65)	0.005	0.53 (0.19, 1.45)	0.217	1.10 (0.77, 1.56)	0.596		1.63 (1.27, 2.09)	<0.001
**Birthweight (grams)**									
Low (< 2,500)	1.37 (1.14, 1.63)	0.001	3.54 (1.93, 6.49)	<0.001	1.21 (0.85, 1.71)	0.286		1.29 (1.01, 1.64)	0.040
Normal (2,500 to 3,999)	1.0	-	1.0	-	1.0	-		1.0	-
High (≥ 4,000)	0.77 (0.64, 0.91)	0.003	0.62 (0.30, 1.27)	0.191	0.90 (0.67, 1.22)	0.502		0.70 (0.55, 0.88)	0.002
Not weighed at birth	1.06 (0.96, 1.18)	0.242	1.17 (0.73, 1.87)	0.523	1.03 (0.85, 1.26)	0.747		1.09 (0.95, 1.25)	0.231
Missing	1.15 (0.95, 1.40)	0.148	1.61 (0.59, 4.41)	0.352	1.09 (0.76, 1.58)	0.635		1.06 (0.85, 1.33)	0.589
**Maternal age, years**									
15 to 19	1.33 (1.04, 1.69)	0.023	0.48 (0.18, 1.29)	0.147	1.03 (0.68, 1.57)	0.878	2.11 (1.56, 2.86)		<0.001
20 to 24	1.27 (1.05, 1.53)	0.013	1.44 (0.60, 3.42)	0.413	0.99 (0.71, 1.37)	0.942	1.67 (1.34, 2.08)		<0.001
25 to 29	1.08 (0.92, 1.26)	0.367	1.13 (0.45, 2.83)	0.796	0.88 (0.66, 1.17)	0.378	1.29 (1.08, 1.55)		0.005
30 to 34	1.10 (0.95, 1.27)	0.188	1.21 (0.61, 2.39)	0.579	1.04 (0.80, 1.35)	0.797	1.22 (1.03, 1.44)		0.025
35 and older	1.0	-	1.0	-	1.0	-	1.0		-
**Maternal education,**									
No formal education	1.0	-	1.0	-	1.0	-	1.0		-
Primary (grades 1 to 6)	0.98 (0.89, 1.08)	0.698	0.75 (0.47, 1.20)	0.228	0.95 (0.79, 1.13)	0.533	1.03 (0.91, 1.16)		0.683
Secondary (grades 7 to 12)	0.79 (0.67, 0.94)	0.009	0.66 (0.33, 1.31)	0.232	0.92 (0.69, 1.24)	0.599	0.75 (0.60, 0.93)		0.008
Higher	0.36 (0.19, 0.68)	0.002	0.60 (0.14, 2.60)	0.492	0.23 (0.06, 0.82)	0.023	0.29 (0.14, 0.62)		0.001
**Antenatal care, visits**									
Less than four (< 4)	1.10 (0.97, 1.25)	0.122	0.94 (0.62, 1.43)	0.769	1.11 (0.94, 1.32)	0.218	1.18 (0.97, 1.43)		0.090
Four of more (≥ 4)	1.0	-	1.0	-	1.0	-	1.0		-
Missing	1.14 (0.74, 1.74)	0.556	3.66 (1.05, 12.8)	0.042	1.36 (0.74, 2.50)	0.314	0.89 (0.46, 1.72)		0.731
**Newborn health visit**									
No	1.0	-	1.0	-	1.0	-	1.0		-
Yes	1.03 (0.90, 1.18)	0.661	1.24 (0.68, 2.25)	0.487	1.04 (0.85, 1.27)	0.722	1.02 (0.83, 1.25)		0.863
Missing	0.89 (0.58, 1.35)	0.575	0.65 (0.17, 2.58)	0.543	0.96 (0.54, 1.73)	0.900	1.04 (0.55, 1.96)		0.914
**Breastfeeding duration**									
Currently	1.0	-	1.0	-	1.0	-	1.0		-
Not currently	1.49 (1.33, 1.66)	<0.001	0.87 (0.42, 1.79)	0.700	1.19 (1.00, 1.42)	0.045	0.86 (0.62, 1.19)		0.371
Never breastfed	1.43 (1.16, 1.77)	0.001	1.21 (0.59, 2.49)	0.607	1.07 (0.76, 1.51)	0.692	0.93 (0.66, 1.30)		0.662
**Diarrhea in last 2 weeks**									
No	1.0	-	1.0	-	1.0	-	1.0		-
Yes	1.30 (1.17, 1.45)	<0.001	0.42 (0.17, 1.08)	0.073	1.23 (1.05, 1.44)	0.011	1.34 (1.15, 1.57)		<0.001
**Work outside of home**									
Working	1.0	-	1.0	-	1.0	-	1.0		-
Not working	0.93 (0.80, 1.07)	0.279	0.96 (0.59, 1.58)	0.877	0.92 (074, 1.14)	0.447	0.95 (0.80, 1.11)		0.503
**Paternal age, years**									
15 to 19	0.94 (0.61, 1.47)	0.798	10.5 (3.53, 31.1)	<0.001	0.78 (0.35, 1.78)	0.559	0.74 (0.34, 1.61)		0.447
20 to 24	1.25 (1.01, 1.54)	0.041	1.28 (0.49, 3.37)	0.614	1.51 (1.13, 2.01)	0.005	1.18 (0.89, 1.56)		0.240
25 to 29	1.12 (0.94, 1.33)	0.215	0.73 (0.30, 1.78)	0.483	1.34 (1.02, 1.77)	0.033	1.04 (0.84, 1.29)		0.717
30 to 34	1.08 (0.91, 1.27)	0.400	1.43 (0.61, 3.33)	0.411	1.27 (0.97, 1.66)	0.088	0.93 (0.78, 1.11)		0.431
35 and older	1.0	-	1.0	-	1.0	-	1.0		-
Missing	0.86 (0.67, 1.10)	0.229	0.87 (0.20, 3.85)	0.856	0.81 (0.48, 1.39)	0.446	0.86 (0.63, 1.18)		0.353
**Paternal education,**									
No formal education	1.0	-	1.0	-	1.0	-	1.0		-
Primary	0.94 (0.83, 1.07)	0.370	2.00 (1.17, 3.43)	0.011	0.95 (0.74, 1.21)	0.676	0.89 (0.76, 1.03)		0.123
Secondary	0.89 (0.76, 1.04)	0.157	1.19 (0.62, 2.30)	0.596	0.84 (0.65, 1.08)	0.177	0.88 (0.73, 1.06)		0.190
Higher	0.62 (0.39, 0.97)	0.038	1.13 (0.35, 3.66)	0.840	0.56 (0.27, 1.20)	0.137	0.62 (0.33, 1.16)		0.134
Missing	0.89 (0.73, 1.09)	0.269	0.65 (0.29, 1.47)	0.301	1.10 (0.82, 1.47)	0.519	0.84 (0.64, 1.09)		0.185
**Source of water**									
Piped	1.0	-	1.0	-	1.0	-	1.0		-
Fountain, well	1.30 (1.06, 1.60)	0.011	2.51 (1.16, 5.44)	0.019	1.28 (0.97, 1.70)	0.079	1.24 (0.98, 1.58)		0.070
Spring/ surface/ rain	1.33 (1.07, 1.65)	0.011	2.00 (0.85, 4.68)	0.110	1.29 (0.95, 1.73)	0.100	1.29 (1.01, 1.66)		0.043
Other	1.11 (0.90, 1.38)	0.337	2.33 (1.07, 5.05)	0.032	1.35 (0.96, 1.92)	0.087	0.96 (0.73, 1.26)		0.758
**Sanitary system**									
Septic tank	1.0	-	1.0	-	1.0	-	1.0		-
Public sanitary sewer	1.24 (0.99, 1.56)	0.066	0.84 (0.36, 1.99)	0.699	1.26 (0.88, 1.79)	0.203	1.26 (0.95, 1.69)		0.111
Open pit	1.22 (1.06, 1.41)	0.006	0.94 (0.51, 1.75)	0.852	1.22 (0.99, 1.52)	0.068	1.31 (1.08, 1.59)		0.006
No sanitary facility	1.05 (0.91, 1.20)	0.522	0.51 (0.29, 0.92)	0.025	1.03 (0.83, 1.28)	0.799	1.08 (0.91, 1.28)		0.397
Other	1.23 (0.89, 1.70)	0.217	1.00 (0.23, 4.30)	0.997	0.46 (0.12, 1.81)	0.266	1.70 (1.24, 2.35)		0.001
**Electricity**									
Yes	1.0	-	1.0	-	1.0	-	1.0		-
No	1.04 (0.88, 1.23)	0.634	1.02 (0.56, 1.87)	0.945	1.02 (0.79, 1.31)	0.868	1.01 (0.84, 1.21)		0.916
**Refrigerator**									
Yes	1.0	-	1.0	-	1.0	-	1.0		-
No	1.46 (1.18, 1.81)	0.001	2.90 (0.89, 9.44)	0.077	1.87 (1.24, 2.81)	0.003	1.16 (0.90, 1.49)		0.255
**Eligible children in household**									
One	0.87 (0.78, 0.98)	0.027	0.69 (0.39, 1.20)	0.183	0.80 (0.64, 0.98)	0.035	0.86 (0.72, 1.02)		0.082
Two	1.0	-	1.0	-	1.0	-	1.0		-
Three or more	0.93 (0.84, 1.02)	0.124	1.18 (0.74, 1.87)	0.480	0.90 (0.76, 1.08)	0.279	1.16 (0.90, 1.49)		0.255
**Wealth Index**									
Poorest	0.99 (0.80, 1.23)	0.950	1.75 (0.64, 4.78)	0.272	1.29 (0.87, 1.92)	0.200	0.91 (0.71, 1.16)		0.455
Poorer	0.94 (0.79, 1.13)	0.514	1.53 (0.66, 3.52)	0.322	1.13 (0.82, 1.56)	0.453	0.88 (0.72, 1.07)		0.208
Middle	1.0	-	1.0	-	1.0	-	1.0		-
Wealthier	1.03 (0.82, 1.30)	0.784	1.36 (0.44, 4.22)	0.590	1.05 (0.72, 1.53)	0.809	0.91 (0.69, 1.19)		0.470
Wealthiest	1.06 (0.72, 1.54)	0.778	3.30 (0.72, 15.1)	0.123	0.88 (0.44, 1.77)	0.720	0.91 (0.61, 1.35)		0.631
**Area of residence**									
Urban	1.0	-	1.0	-	1.0	-	1.0		-
Rural	0.98 (0.87, 1.11)	0.774	1.24 (0.69, 2.26)	0.473	1.15 (0.90, 1.47)	0.271	0.89 (0.77, 1.03)		0.117

^1^Fully adjusted multivariable Poisson regression model (model 3) was used to obtain prevalence rations. Values reported are adjusted prevalence-ratio (aPR), 95% confidence interval (95% CI), and p-value of the hypothesis test that prevalence of each level of exposure equals the prevalence for the baseline level of variable.

### Multilevel mixed-effects analysis

Prevalence ratios, confidence intervals, and hypothesis testing p-values obtained using multilevel, mixed-effects models were similar to those estimated with fixed-effects models (S3 Table). Exposures associated with stunting in the fixed-effects model were also associated with stunting in multilevel mixed-models (S3 Table). Variables where estimates differed between mixed and fixed effects were birthweight, cohabitation, ANC visits, paternal age, source of drinking water, sanitary system, and type of cooking fuel (S3 Table). Association of those variables with risk of stunting was stronger in the mixed-effects models, with either a larger effect size, or narrower confidence interval. The only exception was source of drinking water, where adjusting for clustering of observation decreased effect size (S3 Table).

## Discussion

### Main results

This is the first study to describe factors associated with stunting among children 0 to 59 months age in Angola using nationally representative data. Prevalence of stunting is high among children 0 to 59 months of age in Angola, and exposures associated with stunting are consistent with known risk factors for stunting in LMICs [27]. Male children, after the first year of life, with low birthweight, or experiencing frequent episodes of diarrhea are at higher risk for stunting. Young mothers, with low educational level, widowed, divorced or separated, or who did not receive recommended minimum ANC are also more likely to have children with stunted growth. Effects of paternal age and education are weaker than those observed for maternal age and education, but risk of stunting was higher for children of younger fathers, and lower when fathers reached higher education. Source of water was strongly associated with stunting. An unexpected finding was that children residing in households connected to public sewer were at increased risk for stunting, even after accounting for clustering of observations. It is unclear why access to better sanitation would be associated with higher risk of stunting. Possible explanations include residual confounding by unmeasured variables, and improper disposal of raw sewage into local streams or coastal areas rendering public sewer harmful to local residents [41–44]. Food preservation seems to help prevent stunting, since access to refrigerator reduces risk of stunting. Lower risk of stunting among children in households cooking with charcoal, relative to households cooking with liquified petroleum gas (LPG), may also be due to residual confounding, since sources of indoor air-pollution are usually associated with adverse consequences to child health [45]. Lastly, the results reveal the existence of variables operating at the provincial level to determine the risk of stunting, even after adjusting to a broad range of individual and household-level variables. The existence of area-level variables is aligned with the conceptual framework on the causes of stunting, which recognizes distal factors as important determinants of child nutrition and growth [4, 29, 38]. It is important to notice that the effects of exposures were not homogeneous across age-groups. Effect modification by child age-group was particularly noticeable for birth order. First-born children were at much greater risk for stunting during the first six months of life, but the opposite effect was observed for those older than 24 months. This seems to indicate that parental experience is an important determinant of nutrition and growth for younger children, while older children were more vulnerable when born later into the family, possibly indicating changes in parenting behavior in families with multiple children. As expected, children with low birthweight were more vulnerable to stunting early in life. Younger children also appeared to be more sensitive to contamination of water and food, with water source and food preservation (measured as access to refrigeration) showing stronger effects among children 0 to 5 months of age. Stratified analysis also produced unexpected finding; exposures expected to have larger effects on the risk of stunting among younger children, completion of antenatal care, newborn health visit, and breastfeeding, did not show any association with stunting among children 0 to 5 months of age. Rather than a lack of independent effect, these results might be due to insufficient sample size. In the case of antenatal care, adjustment for birthweight likely account for the benefits that ANC completion might have on risk of stunting. Those examples underscore the complex interaction of exposures that influence risk of stunting at specific periods in the first five years of child life.

### Strengths and limitations

Several characteristics of this study support internal validity, including complex, multistage, probabilistic sampling, use of validated questionnaires and standardized techniques to ascertain exposures and outcome, training of staff, and use of multivariable regression analysis to adjust for confounding. Quality of anthropometric data was confirmed by the standard deviation of height-for-age Z-score (SD = 1.52) within expected range (1.35 to 1.95) [46]. Main limitations of this study are the possibility of residual confounding by unmeasured exposures, cross-sectional design of the study, which creates opportunity for survival bias, and the limited capacity to investigate effect modification by child age, as the study was not adequately powered to support analysis stratified by child age-group. Effect of maternal exposure variables on child growth and stunting may be confounded by early life exposures experienced by mothers, particularly for mothers who experienced stunting. Absence of information on maternal height is a particularly important source of residual confounding, since maternal short stature is an independent risk factor for stunting [27]. Cross-sectional design of this study does not allow to determine if the effect of exposures is on incidence or duration of stunting. In addition, survival bias is of particular concern in the setting of LMICs such as Angola, where child mortality is high. In the case of this study, proportion of children by age-group decreased over the first five years of life (S2 Table), from about 24% for children under one year of age, to 17% for those in the fifth year of life, reflecting the high under-five mortality rate in Angola (75 per 1,000 children-year) [47]. Stunted children are likely to experience higher mortality, relative to children with normal height-for-age, lowering the prevalence of stunting estimated in a cross-sectional study [48]; consequently, survival bias may weaken the effect of risk factors for stunting estimated in a cross-sectional study [49]. Despite the possibility of survival bias, this study was able to detect important risk factors for stunting among children under 5 years of age in Angola. Lastly, it is well described that growth faltering develops during the first 23 months of life [50], and that the risk factors for stunting change over the course of the first years of life [51]. Ability to detect age-specific effects of exposures using stratified analysis was partially limited by sample size available and lower prevalence of stunting during in the 0 to 5 and 6 to 23 months of age groups. Nevertheless, important differences in the effect of exposure variables according to age-group were identified.

### Interpretation

Risk factors for stunting in Angola identified here are aligned with the framework on causation of malnutrition and stunting described in the literature [2, 4, 29] and are similar to those reported for other countries [27, 28]. Strategies and interventions to reduce stunting described in the literature [50, 52] may also work in Angola. Our results indicate that delaying pregnancy, decreasing parity, raising maternal educational level, increasing birth weight, and preventing and treating diarrhea may lower the prevalence of stunting in Angola. Delaying age of fatherhood and improving paternal education may also help, although the effects are not as robust. Ensuring access to clean water, sanitation, and food refrigeration can also contribute to decreasing prevalence of stunting. Contextual factors within provinces, not captured in exposures ascertained at individual and household level, also appear to influence risk of stunting. Lastly, our findings are in alignment with the idea that interventions to prevent stunting need to be delivered early on in the life of children at-risk, since prevalence of stunting increases rapidly in the first two years of life [50].

### Generalizability

Findings from this study are applicable to LMICs with similar demographic structure, and socio-cultural, geographic, and climatic characteristics, and are particularly relevant to southern Africa. The exposures associated with stunting in this study are in agreement with findings from DHS studies in other countries [27], further supporting the generalizability of results. However, exposures associated with stunting at the national level may not be applicable to individual provinces. Local conditions in the provinces may influence and modify the effect of individual and household-level exposures on child growth.

## Conclusions

This is the first study to report on a comprehensive set of risk and protective factors for stunting among children under five years of age in Angola using a nationally representative sample. Children in Angola are at high risk for stunted growth, even relative to countries in the same region [1]. Angola shares the same risk factors for stunting that have been described in other developing countries [27, 28]. Maternal education, reproductive health, prevention and treatment of diarrhea, water and sanitation infrastructure, electrification of households and access to food refrigeration are potential targets for programs aiming to reduce prevalence of stunting in Angola.

## Supporting information

S1 Appendix Sample size calculation. Calculation of the relative standard error (RSE) and sample size needed to estimate prevalence of stunting with a 2.5% margin of error.
(DOCX)

S1 Table Sample size required according to magnitude of effect (odds-ratio) to be detected. Sample sizes required to detect a pre-specified magnitude of effect were calculated for a range of expected odds-ratio (OR), assuming a power of 0.8, alpha of 0.05, prevalence of outcome (stunting) of 38.8% in the reference group, and two exposure groups of equal size.
(DOCX)

S2 Table Characteristics of children, parents, and households for children selected for anthropometry and included in final analysis. Values are weighted counts of children and row percentage (%).
(DOCX)

S3 Table Prevalence ratio according to characteristics of participants and households adjusted (aPR) for covariates using multilevel, mixed-effects, multivariable Poisson regression. Values reported are prevalence ratios adjusted for confounding and clustering of observations using multilevel, mixed-effects Poisson regression (aPR), 95% confidence interval (95% CI), and p-value of the hypothesis test that prevalence of each level of exposure equals the prevalence for the baseline level of variable.
(DOCX)

S4 Table Crude and adjusted absolute risk difference of stunting according to characteristics of participants and households. Value reported are crude absolute risk difference (ARD) and absolute risk difference adjusted for confounding using multivariable, fixed-effects, Poisson regression (aARD), 95% confidence interval (95% CI), and p-value of the hypothesis test that prevalence of each level of exposure equals the prevalence for the baseline level of variable.
(DOCX)

S1 Fig Sample selection in the Angola 2015–2016 Demographic and Health Survey (DHS). Flowchart describing number of clusters (primary sampling units), households, women, and children selected for participation, number excluded with reason for exclusion, and number of participants included in study sample.
(TIF)

S2 Fig Prevalence of stunting according to child age. Graph shows the prevalence of stunting expressed as proportion with 95% confidence interval (95% CI), according to child age-group categorized in 6-month intervals.
(TIF)
