# Supplementary material for: Factors associated with stunting among children 0 to 59 months of age in Angola: A cross-sectional study using the 2015–2016 Demographic and Health Survey
Source: PLOS Glob Public Health. 2022 Dec 12;2(12):e0000983. doi: 10.1371/journal.pgph.0000983 (PMC10021435; doi:10.1371/journal.pgph.0000983)
Supplement: S1 Appendix — Calculation of the relative standard error (RSE) and sample size needed to estimate prevalence of stunting with a 2.5% margin of error. (DOCX) [file pgph.0000983.s001.docx]

## S1 Appendix. Sample size calculation to estimate prevalence of stunting with sufficient precision.

Sample size was calculated to estimate the prevalence of stunting with a 2.5% margin of error, assuming two distinct design effects, 1.5 and 1.8 [[1](#_ENREF_1)], and an expected prevalence of 38.8% [[2](#_ENREF_2)]. Width of the confidence interval is determined by the relative standard error (RSE), which describes the amount of sampling error relative to the estimate of the variable of interest, and is independent of the scale of the variable to be estimated [[3](#_ENREF_3)]. For a confidence level of 95%, the half-length of the confidence interval for an estimate of proportion p can be estimated with the formula [[3](#_ENREF_3)]:

$margin of error=2\times p\times RSE$ ( 1 )

For a margin of error of 2.5% and expected prevalence of 39% [[2](#_ENREF_2)]

$0.025=2\times0.39\times RSE$ ( 2 )

$RSE=\frac{0.025}{0.78}$ ( 3 )

$RSE=0.0321$ ( 4 )

$RSE=0.03$ ( 5 )

Where p represents the prevalence of outcome, n_c_ represents the sample size for clustered design, and d represents the design effect (deff), n_c_ can be calculated using the formula [[3](#_ENREF_3)]:

$n_{c}=d^{2}\times\frac{\left( 1-p \right)}{\left( {RSE}_{p} \right)^{2}\times p}$ ( 1 )

Assuming the expected prevalence of stunting to be 38.8% [[2](#_ENREF_2)], and a design effect of 1.5 [[1](#_ENREF_1)], the following values as substituted on the formula:

$d=1.5$; $p=0.388$; $\left( 1-p \right)=0.612$; $\mathrm{RSE}_{p}=0.03$.

$n_{c}=\left( 1.5 \right)^{2}\times\frac{\left( 0.612 \right)}{\left( 0.03 \right)^{2}\times0.388}$ ( 2 )

$n_{c}=\frac{\left( 1.5 \right)^{2}\times0.612}{\left( 0.03 \right)^{2}\times0.388}$ ( 3 )

$n_{c}=\frac{2.25\times6.12\times{10}^{-1}}{9\times{10}^{-4}\times3.88\times{10}^{-1}}$ ( 4 )

$n_{c}=3,943$ ( 5 )

Therefore, the number of children 0- to 59-month-old to be sampled from the target population to estimate prevalence of stunting of 38.8%, given a design effect (deff) of 1.5 [[1](#_ENREF_1)] due to cluster sampling is 3,943. For a design effect of 1.8 [[1](#_ENREF_1)], the number is 5,678 children.

1. Hulland EN, Blanton CJ, Leidman EZ, Bilukha OO. Parameters associated with design effect of child anthropometry indicators in small-scale field surveys. Emerging Themes in Epidemiology. 2016;13(1):13.

2. Li Z, Kim R, Vollmer S, Subramanian SV. Factors Associated With Child Stunting, Wasting, and Underweight in 35 Low- and Middle-Income Countries. JAMA Network Open. 2020;3(4):e203386-e.

3. ICF International. Demographic and Health Survey Sampling and Household Listing Manual. Calverton, MD, USA: ICF International; 2012.
