# Supplementary material for: Factors associated with stunting among children 0 to 59 months of age in Angola: A cross-sectional study using the 2015–2016 Demographic and Health Survey
Source: PLOS Glob Public Health. 2022 Dec 12;2(12):e0000983. doi: 10.1371/journal.pgph.0000983 (PMC10021435; doi:10.1371/journal.pgph.0000983)
Supplement: S1 Table — Sample sizes required to detect a pre-specified magnitude of effect were calculated for a range of expected odds-ratio (OR), assuming a power of 0.8, alpha of 0.05, prevalence of outcome (stunting) of 38.8% in the reference group, and two exposure groups of equal size. (DOCX) [file pgph.0000983.s002.docx]

**S1 Table. Sample sizes required according to magnitude of effect (odds-ratio) to be detected.**

| **OR** | **Total sample** | **Sample in each group** | **Sample in each group** | |
| --- | --- | --- | --- | --- |
|  |  |  | **Design effect** | |
|  |  |  | **1.5** | **1.8** |
| 1.30 | 1,878 | 939 | 1,409 | 1,690 |
| 1.25 | 2,602 | 1,301 | 1,952 | 2,342^2^ |
| 1.20 | 3,910 | 1,955 | 2,933^1^ | 3,519 |
| 1.15 | 6,676 | 3,338 | 5,007 | 6,008 |
| 1.10 | 14,412 | 7,206 | 10,809 | 12,971 |
| 0.91 | 15,036 | 7,518 | 11,277 | 13,532 |
| 0.87 | 6,938 | 3,469 | 5,204 | 6,244 |
| 0.83 | 3,902 | 1,951 | 2,927^1^ | 3,512 |
| 0.80 | 2,736 | 1,368 | 2,052 | 2,462^2^ |
| 0.77 | 2,006 | 1,003 | 1,505 | 1,805 |
| ^1^Sample size available (total sample n=5,905; n= 2,952 per exposure group) is sufficiently large to detect an odds-ratio equal to or larger than 1.20, or equal to or smaller than 0.83, for a design effect of 1.5.  ^2^Sample size available (total sample n=5,905; n= 2,952 per exposure group) is sufficiently large to detect an odds-ratio equal to or larger than 1.25, or equal to or smaller than 0.80, for a design effect of 1.8. | | | | |
