# Supplementary material for: Factors associated with stunting among children 0 to 59 months of age in Angola: A cross-sectional study using the 2015–2016 Demographic and Health Survey
Source: PLOS Glob Public Health. 2022 Dec 12;2(12):e0000983. doi: 10.1371/journal.pgph.0000983 (PMC10021435; doi:10.1371/journal.pgph.0000983)
Supplement: S2 Table — Values are weighted counts of children and row percentage (%). (DOCX) [file pgph.0000983.s003.docx]

**S2 Table. Characteristics of children, parents, and households for children selected for anthropometry and included in final analysis.**

| **Characteristic** | **Weighted count, number (%)** | |
| --- | --- | --- |
|  | **Selected** | **Analyzed** |
| **Total** | 6,296 (100) | 5,905 (100) |
| **Sex of child** |  |  |
| Male | 3,155 (50.1) | 2,954 (50.0) |
| Female | 3,141 (49.9) | 2,950 (50.0) |
| **Child age-group, months** |  |  |
| 0 to 11 | 1,515 (24.1) | 1,409 (23.9) |
| 12 to 23 | 1,288 (20.5) | 1,215 (20.6) |
| 24 to 35 | 1,197 (19.0) | 1,119 (19.0) |
| 36 to 47 | 1,242 (19.7) | 1,175 (19.9) |
| 48 to 59 | 1,053 (16.7) | 987 (16.7) |
| **Birth order** |  |  |
| First | 1,289 (20.5) | 1,205 (20.4) |
| Second | 1,264 (20.1) | 1,188 (20.1) |
| Third and fourth | 1,909 (30.3) | 1,816 (30.8) |
| Fifth and above | 1,834 (29.1) | 1,696 (28.7) |
| **Maternal age-group, years** |  |  |
| 15 to 19 | 561 (8.9) | 533 (9.0) |
| 20 to 24 | 1,712 (27.2) | 1,597 (27.0) |
| 25 to 29 | 1,637 (26.0) | 1,547 (26.2) |
| 30 to 34 | 1,125 (17.9) | 1,063 (18.0) |
| 35 and older | 1,261 (20.0) | 1,164 (19.7) |
| **Maternal education** |  |  |
| No formal education | 1,873 (29.7) | 1,730 (29.3) |
| Primary | 2,573 (40.9) | 2,443 (41.4) |
| Secondary | 1,653 (26.3) | 1,553 (26.3) |
| Higher | 198 (3.1) | 178 (3.0) |
| **Marital status** |  |  |
| Never in union | 852 (13.5) | 802 (13.6) |
| Married | 990 (15.7) | 912 (15.5) |
| Partner | 3,953 (62.8) | 3,725 (63.1) |
| Widowed, divorced, separated | 501 (8.0) | 465 (7.9) |
| **Cohabitation** |  |  |
| Never in union | 852 (13.5) | 802 (13.6) |
| Living together | 4,457 (70.8) | 4,192 (71.0) |
| Living separated | 486 (7.7) | 446 (7.6) |
| Widowed, divorced, separated | 501 (8.0) | 465 (7.9) |

| S2 Table (continued) | | |
| --- | --- | --- |
| **Characteristic** | **Weighted count, number (%)** | |
|  | **Selected** | **Analyzed** |
| **Work outside of home** |  |  |
| No | 1,640 (26.0) | 1,524 (25.8) |
| Yes | 4,656 (74.0) | 4,380 (74.2) |
| **Age of sexual initiation** |  |  |
| 14 and under | 1,661 (26.4) | 1,542 (26.1) |
| 15 and 16 | 2,400 (38.1) | 2,263 (38.3) |
| 17 and above | 2,235 (35.5) | 2,100 (35.6) |
| **Healthcare decision** |  |  |
| Husband, partner, other | 1,277 (20.3) | 1,199 (20.3) |
| Joint | 2,645 (42.0) | 2,472 (41.9) |
| Woman | 1,021 (16.2) | 966 (16.4) |
| Father not present | 1,353 (21.5) | 1,267 (21.5) |
| **Autonomy to visit family** |  |  |
| Husband, partner, other | 649 (10.3) | 604 (10.2) |
| Joint | 2,889 (45.9) | 2,715 (46.0) |
| Woman | 1,405 (22.3) | 1,319 (22.3) |
| Father not present | 1,353 (21.5) | 1,267 (21.5) |
| **Sexual autonomy** |  |  |
| No, not sure | 1,892 (30.1) | 1,786 (30.2) |
| Yes | 3,051 (48.5) | 2,852 (48.3) |
| Father not present | 1,353 (21.5) | 1,267 (21.5) |
| **Safe-sex autonomy** |  |  |
| No, not sure | 2,264 (35.9) | 2,146 (36.3) |
| Yes | 2,679 (42.6) | 2,492 (42.2) |
| Father not present | 1,353 (21.5) | 1,267 (21.5) |
| **Lifetime natality control** |  |  |
| No | 4,834 (76.8) | 4,560 (77.2) |
| Yes | 1,462 (23.2) | 1,345 (22.8) |
| **Antenatal care, visits** |  |  |
| Less than four (< 4) | 1,491 (23.7) | 1,386 (23.5) |
| Four of more (≥ 4 ) | 2,549 (40.5) | 2,392 (40.5) |
| Missing | 2,256 (35.8) | 2,127 (36.0) |
| **Birthweight, grams** |  |  |
| Low (< 2,500) | 351 (5.6) | 334 (5.7) |
| Normal (2,500 to 3,999) | 2,553 (40.5) | 2,405 (40.7) |
| Large (≥ 4,000) | 638 (10.1) | 598 (10.1) |
| Not weighed at birth | 2,472 (39.3) | 2,317 (39.2) |
| Missing | 282 (4.5) | 251 (4.3) |
| **Newborn health visit** |  |  |
| No | 3,085 (49.0) | 2,886 (48.9) |
| Yes | 952 (15.1) | 891 (15.1) |
| Missing | 2,259 (35.9) | 2,128 (36.0) |

| S2 Table (continued) | | |
| --- | --- | --- |
| **Characteristic** | **Weighted count, number (%)** | |
|  | **Selected** | **Analyzed** |
| **Breastfeeding** |  |  |
| Currently | 2,283 (36.3) | 2,136 (36.2) |
| Not currently | 3,769 (59.9) | 3,545 (60.0) |
| Never breastfed | 245 (3.9) | 224 (3.8) |
| **Diarrhea in last 2 weeks** |  |  |
| No | 5,279 (83.8) | 4,947 (83.8) |
| Yes | 1,005 (16.0) | 947 (16.0) |
| Missing | 13 (0.2) | 11 (0.2) |
| **Fever in last 2 weeks** |  |  |
| No | 5,280 (83.9) | 4,938 (83.6) |
| Yes | 1,009 (16.0) | 960 (16.3) |
| Missing | 8 (0.1) | 7 (0.1) |
| **Cough in last 2 weeks** |  |  |
| No | 5,508 (87.5) | 5,162 (87.4) |
| Yes | 783 (12.4) | 738 (12.5) |
| Missing | 5 (0.1) | 4 (0.1) |
| **Paternal age-group, years** |  |  |
| 15 to 19 | 55 (0.9) | 53 (0.9) |
| 20 to 24 | 498 (7.9) | 477 (8.1) |
| 25 to 29 | 1,112 (17.7) | 1,036 (17.5) |
| 30 to 34 | 985 (15.7) | 928 (15.7) |
| 35 to 84 | 2,179 (34.6) | 2,046 (34.7) |
| Father not present | 1,353 (21.5) | 1,267 (21.5) |
| Missing | 114 (1.8) | 97 (1.6) |
| **Paternal education** |  |  |
| No formal education | 674 (10.7) | 620 (10.5) |
| Primary | 1,619 (25.7) | 1,548 (26.2) |
| Secondary | 1,984 (31.5) | 1,871 (31.7) |
| Higher | 296 (4.7) | 266 (4.5) |
| Father not present | 1,353 (21.5) | 1,267 (21.5) |
| Missing | 370 (5.9) | 334 (5.7) |
| **Source of water** |  |  |
| Piped | 1,457 (23.1) | 1,349 (22.9) |
| Fountain/ well | 2,020 (32.1) | 1,919 (32.5) |
| Spring/ surface/ rain | 1,770 (28.1) | 1,668 (28.3) |
| Other | 999 (15.9) | 920 (15.6) |
| Missing | 50 (0.8) | 49 (0.8) |

| S2 Table (continued) | | |
| --- | --- | --- |
| **Characteristic** | **Weighted count, number (%)** | |
|  | **Selected** | **Analyzed** |
| **Sanitary system** |  |  |
| Public sewage | 394 (6.3) | 373 (6.3) |
| Septic tank | 3,427 (54.4) | 3,210 (54.4) |
| Open pit | 429 (6.8) | 400 (6.8) |
| No sanitation | 1,948 (30.9) | 1,834 (31.1) |
| Other | 48 (0.8) | 39 (0.7) |
| Missing | 50 (0.8) | 49 (0.8) |
| **Shared toilet** |  |  |
| No | 2,757 (43.8) | 2,581 (43.7) |
| Yes | 1,541 (24.5) | 1,442 (24.4) |
| No toilet | 1,948 (30.9) | 1,834 (31.1) |
| Missing | 50 (0.8) | 49 (0.8) |
| **Electricity in household** |  |  |
| No | 3,832 (60.9) | 3,601 (61.0) |
| Yes | 2,414 (38.3) | 2,255 (38.2) |
| Missing | 50 (0.8) | 49 (0.8) |
| **Refrigerator ownership** |  |  |
| No | 4,157 (66.0) | 3,912 (66.3) |
| Yes | 2,089 (33.2) | 1,944 (32.9) |
| Missing | 50 (0.8) | 49 (0.8) |
| **Flooring** |  |  |
| Earth/ sand | 3,236 (51.4) | 3,043 (51.5) |
| Cement | 2,304 (36.6) | 2,155 (36.5) |
| Ceramic/ Stone | 638 (10.1) | 591 (10.0) |
| Wood/ Other | 68 (1.2) | 66 (1.1) |
| Missing | 50 (0.8) | 49 (0.8) |
| **Cooking fuel** |  |  |
| Gas | 2,972 (47.2) | 2,770 (46.9) |
| Charcoal | 954 (15.2) | 913 (15.5) |
| Biomass | 2,131 (33.9) | 1,994 (33.8) |
| Other | 189 (3.0) | 179 (3.0) |
| Missing | 50 (0.8) | 49 (0.8) |
| **Household members, number** |  |  |
| 1 to 4 | 1,591 (25.3) | 1,496 (25.3) |
| 5 to 6 | 2,145 (34.1) | 2,032 (34.4) |
| 7 and above | 2,559 (40.7) | 2,376 (40.2) |
| **Eligible women in household** |  |  |
| One | 4,768 (75.7) | 4,491 (76.1) |
| Two | 1,076(17.1) | 990 (16.8) |
| Three or more | 452 (7.2) | 424 (7.2) |

| S2 Table (continued) | | |
| --- | --- | --- |
| **Characteristic** | **Weighted count, number (%)** | |
|  | **Selected** | **Analyzed** |
| **Eligible children in household** |  |  |
| One | 1,468 (23.3) | 1,364 (23.1) |
| Two | 2,817 (44.7) | 2,637 (44.7) |
| Three or more | 2,011 (31.9) | 1,903 (32.2) |
| **Wealth Index, quintiles** |  |  |
| Poorest | 1,371 (21.8) | 1,291 (21.9) |
| Poorer | 1,456 (23.1) | 1,371 (23.2) |
| Middle | 1,391 (22.1) | 1,316 (22.3) |
| Wealthier | 1,187 (18.9) | 1,107 (18.8) |
| Wealthiest | 891 (14.1) | 820 (13.9) |
| **Area of residence** |  |  |
| Urban | 3,805 (60.4) | 3,550 (60.2) |
| Rural | 2,491 (39.6) | 2,350 (39.8) |
| **Provinces** |  |  |
| Bengo | 88 (1.4) | 82 (1.4) |
| Benguela | 555 (8.8) | 541 (9.2) |
| Bié | 325 (5.2) | 298 (5.0) |
| Cabinda | 132 (2.1) | 126 (2.1) |
| Cuando-Cubango | 102 (1.6) | 94 (1.6) |
| Cuanza Norte | 89 (1.4) | 84 (1.4) |
| Cuanza Sul | 483 (7.7) | 467 (7.9) |
| Cunene | 234 (3.7) | 216 (3.7) |
| Huíla | 581 (9.2) | 548 (9.3) |
| Huambo | 544 (8.7) | 525 (8.9) |
| Luanda | 1,877 (29.8) | 1,717 (29.1) |
| Lunda Norte | 189 (3.0) | 166 (2.8) |
| Lunda Sul | 119 (1.9) | 112 (1.9) |
| Malanje | 276 (4.4) | 263 (4.5) |
| Moxico | 136 (2.2) | 123 (2.1) |
| Namibe | 88 (1.4) | 82 (1.4) |
| Uíge | 357 (5.7) | 345 (5.8) |
| Zaire | 123 (2.0) | 117 (2.0) |
