# Supplementary material for: Factors associated with stunting among children 0 to 59 months of age in Angola: A cross-sectional study using the 2015–2016 Demographic and Health Survey
Source: PLOS Glob Public Health. 2022 Dec 12;2(12):e0000983. doi: 10.1371/journal.pgph.0000983 (PMC10021435; doi:10.1371/journal.pgph.0000983)
Supplement: S3 Table — Values reported are prevalence ratios adjusted for confounding and clustering of observations using multilevel, mixed-effects Poisson regression (aPR), 95% confidence interval (95% CI), and p-value of the hypothesis test that prevalence of each level of exposure equals the prevalence for the baseline level of variable. (DOCX) [file pgph.0000983.s004.docx]

**S3 Table. Prevalence ratio according to characteristics of participants and households adjusted (aPR) for covariates using multilevel, mixed-effects, multivariable Poisson regression.**

| **Characteristic** | **Model 1** | | **Model 2** | | **Model 3** | |
| --- | --- | --- | --- | --- | --- | --- |
|  | **aPR**  **(95% CI)** | **p-value** | **aPR**  **(95% CI)** | **p-value** | **aPR**  **(95% CI)** | **p-value** |
| **Sex of child** |  |  |  |  |  |  |
| Female | 1.0 | - | 1.0 | - | 1.0 | - |
| Male | 1.21  (1.11, 1.31) | <0.001 | 1.21  (1.11, 1.33) | <0.001 | 1.21  (1.11, 1.32) | <0.001 |
| **Child age, months** |  |  |  |  |  |  |
| 0 to 11 | 1.0 | - | 1.0 | - | 1.0 | - |
| 12 to 23 | 2.15  (1.93, 2.39) | <0.001 | 2.15  (1.91, 2.42) | <0.001 | 2.12  (1.89, 2.38) | <0.001 |
| 24 to 35 | 2.44  (2.11, 2.83) | <0.001 | 2.45  (1.95, 3.06) | <0.001 | 2.43  (1.96, 3.01) | <0.001 |
| 36 to 47 | 2.12  (1.80, 2.49) | <0.001 | 2.12  (1.73, 2.61) | <0.001 | 2.15  (1.76, 2.63) | <0.001 |
| 48 to 59 | 1.64  (1.36, 1.99) | <0.001 | 1.66  (1.41, 1.95) | <0.001 | 1.67  (1.41, 1.98) | <0.001 |
| **Birth order** |  |  |  |  |  |  |
| First | 1.0 | - | 1.0 | - | 1.0 | - |
| Second | 1.01  (0.96, 1.08) | 0.633 | 1.02  (0.95, 1.09) | 0.610 | 1.03  (0.97, 1.10) | 0.357 |
| Third and fourth | 1.21  (1.02, 1.43) | 0.025 | 1.22  (1.03, 1.45) | 0.022 | 1.23  (1.04, 1.45) | 0.017 |
| Fifth and above | 1.31  (1.12, 1.54) | 0.001 | 1.34  (1.14, 1.57) | <0.001 | 1.34  (1.16, 1.55) | <0.001 |
| **Birthweight (grams)** |  |  |  |  |  |  |
| Low (< 2,500) | - | - | - | - | 1.33  (1.08, 1.63) | 0.008 |
| Normal (2,500 to 3,999) | - | - | - | - | 1.0 | - |
| High (≥ 4,000) | - | - | - | - | 0.74  (0.65, 0.86) | <0.001 |
| Not weighed at birth | - | - | - | - | 1.10  (0.99, 1.21) | 0.067 |
| Missing | - | - | - | - | 1.18  (1.07, 1.30) | 0.001 |
| **Diarrhea in last 2 weeks** |  |  |  |  |  |  |
| Yes | - | - | - | - | 1.23  (1.13, 1.34) | <0.001 |
| No | - | - | - | - | 1.0 | - |
| Missing | - | - |  | - | 0.24  (0.11, 0.52) | <0.001 |

| S3 Table (continued). | | | | | | |
| --- | --- | --- | --- | --- | --- | --- |
| **Characteristic** | **Model 1** | | **Model 2** | | **Model 3** | |
|  | **aPR**  **(95% CI)** | **p-value** | **aPR**  **(95% CI)** | **p-value** | **aPR**  **(95% CI)** | **p-value** |
| **Fever in last 2 weeks** |  |  |  |  |  |  |
| Yes | - | - | - | - | 0.98  (0.90, 1.08) | 0.715 |
| No | - | - | - |  | 1.0 | - |
| Missing | - | - | - | - | 3.41  (0.84, 13.8) | 0.086 |
| **Cough in last two weeks** |  |  |  |  |  |  |
| Yes | - | - | - | - | 0.98  (0.85, 1.12) | 0.761 |
| No | - | - | - |  | 1.0 | - |
| Missing | - | - | - | - | 0.71  (0.13, 3.80) | 0.689 |
| **Maternal age, years** |  |  |  |  |  |  |
| 15 to 19 | 1.29  (1.05, 1.59) | 0.014 | 1.37  (1.10, 1.71) | 0.005 | 1.33  (1.07, 1.66) | 0.009 |
| 20 to 24 | 1.27  (1.09, 1.49) | 0.003 | 1.32  (1.13, 1.53) | <0.001 | 1.27  (1.09, 1.47) | 0.002 |
| 25 to 29 | 1.08  (0.90, 1.28) | 0.415 | 1.11  (0.94, 1.31) | 0.223 | 1.09  (0.93, 1.29) | 0.292 |
| 30 to 34 | 1.07  (0.94, 1.21) | 0.299 | 1.08  (0.96, 1.21) | 0.209 | 1.09  (0.97, 1.22) | 0.139 |
| 35 and older | 1.0 | - | 1.0 | - | 1.0 | - |
| **Maternal education,** |  |  |  |  |  |  |
| No formal education | 1.0 | - | 1.0 | - | 1.0 | - |
| Primary (grades 1 to 6) | 0.94  (0.88, 1.00) | 0.040 | 0.95  (0.88, 1.02) | 0.142 | 0.96  (0.90, 1.03) | 0.254 |
| Secondary (grades 7 to 12) | 0.75  (0.60, 0.94) | 0.013 | 0.76  (0.59, 0.99) | 0.040 | 0.78  (0.61, 1.00) | 0.054 |
| Higher | 0.36  (0.26, 0.49) | <0.001 | 0.35  (0.25, 0.49) | <0.001 | 0.36  (0.25, 0.52) | <0.001 |
| **Cohabitation status** |  |  |  |  |  |  |
| Living together | 1.0 | - | 1.0 | - | 1.0 | - |
| Living separated | 1.05  (0.93, 1.20) | 0.454 | 1.02  (0.89, 1.18) | 0.739 | 1.02  (0.89, 1.17) | 0.763 |
| Widowed, divorced | 1.09  (0.91, 1.30) | 0.113 | 1.11  (1.00, 1.24) | 0.050 | 1.09  (1.00, 1.20) | 0.044 |
| Never in a union | 1.09  (0.91, 1.29) | 0.139 | 1.12  (0.99, 1.27) | 0.076 | 1.10  (0.96, 1.25) | 0.179 |
| **First sexual activity, age in years** |  |  |  |  |  |  |
| 14 and younger | - | - | 0.93  (0.86, 1.01) | 0.087 | 0.94  (0.87, 1.03) | 0.168 |
| 15 to 16 | - | - | 0.98  (0.90, 1.07) | 0.687 | 0.98  (0.91, 1.07) | 0.699 |
| 17 and older | - | - | 1.0 | - | 1.0 | - |
| S3 Table (continued). | | | | | | |
| **Characteristic** | **Model 1** | | **Model 2** | | **Model 3** | |
|  | **aPR**  **(95% CI)** | **p-value** | **aPR**  **(95% CI)** | **p-value** | **aPR**  **(95% CI)** | **p-value** |
| **Sexual autonomy** | - |  |  |  |  |  |
| Yes | - | - | 1.0 | - | 1.0 | - |
| No | - | - | 1.01  (0.89, 1.15) | 0.883 | 1.02  (0.89, 1.16) | 0.820 |
| **Safe sex autonomy** | - |  |  |  |  |  |
| Yes | - | - | 1.0 | - | 1.0 | - |
| No | - | - | 0.99  (0.91, 1.07) | 0.808 | 0.97  (0.90, 1.06) | 0.524 |
| **Lifetime natality control** |  |  |  |  |  |  |
| Yes | - | - | 1.0 | - | 1.0 | - |
| No | - | - | 1.05  (0.93, 1.18) | 0.409 | 1.06  (0.94, 1.20) | 0.362 |
| **Antenatal care, visits** |  |  |  |  |  |  |
| Less than four (< 4) | - | - | 1.13  (1.07, 1.20) | <0.001 | 1.11  (1.04, 1.18) | 0.002 |
| Four of more (≥ 4) | - | - | 1.0 | - | 1.0 | - |
| Missing | - | - | 1.26  (0.83, 1.90) | 0.285 | 1.18  (0.77, 1.82) | 0.451 |
| **Newborn health visit** |  |  |  |  |  |  |
| No | - | - | 1.0 | - | 1.0 | - |
| Yes | - | - | 1.07  (0.94, 1.22) | 0.279 | 1.04  (0.91, 1.18) | 0.591 |
| Missing | - | - | 0.84  (0.53, 1.31) | 0.438 | 0.87  (0.56, 1.41) | 0.610 |
| **Breastfeeding duration** |  |  |  |  |  |  |
| Currently | - | - | 1.0 | - | 1.0 | - |
| Not currently | - | - | 1.02  (0.87, 1.20) | 0.834 | 1.02  (0.87, 1.20) | 0.782 |
| Never breastfed | - | - | 1.10  (0.97, 1.26) | 0.136 | 1.10  (0.95, 1.27) | 0.206 |
| **Healthcare decision** |  |  |  |  |  |  |
| Joint | - | - | 1.0 | - | 1.0 | - |
| Husband/ partner/ other | - | - | 0.98  (0.86, 1.10) | 0.712 | 0.98  (0.85, 1.12) | 0.721 |
| Woman | - | - | 1.12  (0.94, 1.33) | 0.215 | 1.11  (0.91, 1.35) | 0.313 |
| **Decision to visit family** |  |  |  |  |  |  |
| Joint | - | - | 1.0 | - | 1.0 | - |
| Husband/ partner/ other | - | - | 0.98  (0.84, 1.15) | 0.844 | 0.96  (0.82, 1.13) | 0.659 |
| Woman | - | - | 1.06  (0.97, 1.15) | 0.229 | 1.04  (0.96, 1.12) | 0.326 |
| S3 Table (continued). | | | | | | |
| **Characteristic** | **Model 1** | | **Model 2** | | **Model 3** | |
|  | **aPR**  **(95% CI)** | **p-value** | **aPR**  **(95% CI)** | **p-value** | **aPR**  **(95% CI)** | **p-value** |
| **Work outside of home** |  |  |  |  |  |  |
| Not working | - | - | 0.94  (0.86, 1.02) | 0.127 | 0.94  (0.87, 1.02) | 0.162 |
| Working | - | - | 1.0 | - | 1.0 | - |
| **Paternal age, years** |  |  |  |  |  |  |
| 15 to 19 | 1.06  (0.81, 1.39) | 0.677 | 1.06  (0.81, 1.38) | 0.676 | 1.05  (0.81, 1.37) | 0.702 |
| 20 to 24 | 1.29  (1.04, 1.60) | 0.019 | 1.31  (1.05, 1.64) | 0.018 | 1.30  (1.04, 1.63) | 0.022 |
| 25 to 29 | 1.12  (0.99, 1.26) | 0.071 | 1.12  (0.99, 1.26) | 0.071 | 1.12  (0.99, 1.26) | 0.073 |
| 30 to 34 | 1.09  (0.99, 1.20) | 0.084 | 1.09  (0.99, 1.19) | 0.088 | 1.10  (1.01, 1.19) | 0.034 |
| 35 and older | 1.0 | - | 1.0 | - | 1.0 | - |
| Missing | 0.88  (0.72, 1.07) | 0.186 | 0.87  (0.72, 1.06) | 0.167 | 0.87  (0.72, 1.06) | 0.160 |
| **Paternal education,** |  |  |  |  |  |  |
| No formal education | 1.0 | - | 1.0 | - | 1.0 | - |
| Primary | 0.99  (0.89, 1.10) | 0.846 | 0.98  (0.89, 1.08) | 0.705 | 0.97  (0.87, 1.07) | 0.529 |
| Secondary | 0.92  (0.83, 1.02) | 0.108 | 0.91  (0.81, 1.02) | 0.104 | 0.91  (0.80, 1.02) | 0.099 |
| Higher | 0.64  (0.42, 0.99) | 0.044 | 0.63  (0.40, 1.02) | 0.058 | 0.62  (0.39, 0.98) | 0.040 |
| Missing | 0.92  (0.78, 1.08) | 0.284 | 0.90  (0.76, 1.06) | 0.203 | 0.91  (0.77, 1.06) | 0.228 |
| **Source of water** |  |  |  |  |  |  |
| Piped | 1.0 | - | 1.0 | - | 1.0 | - |
| Fountain, well | 1.23  (0.94, 1.61) | 0.137 | 1.23  (0.96, 1.60) | 0.107 | 1.23  (0.96, 1.58) | 0.106 |
| Spring/ surface/ rain | 1.25  (1.00, 1.58) | 0.054 | 1.26  (1.01, 1.56) | 0.037 | 1.26  (1.02, 1.57) | 0.034 |
| Other | 1.13  (0.97, 1.31) | 0.128 | 1.11  (0.96, 1.29) | 0.171 | 1.14  (0.99, 1.32) | 0.071 |
| Missing | 1.13  (0.64, 1.99) | 0.667 | 1.13  (0.62, 2.04) | 0.696 | 1.18  (0.69, 2.01) | 0.554 |
| **Sanitary system** |  |  |  |  |  |  |
| Septic tank | 1.0 | - | 1.0 | - | 1.0 | - |
| Public sanitary sewer | 1.16  (1.04, 1.30) | 0.009 | 1.17  (1.05, 1.31) | 0.005 | 1.22  (1.07, 1.39) | 0.002 |
| Open pit | 1.18  (1.04, 1.34) | 0.010 | 1.19  (1.06, 1.34) | 0.003 | 1.18  (1.04, 1.34) | 0.008 |
| No sanitary facility | 1.01  (0.90, 1.14) | 0.854 | 1.01  (0.88, 1.15) | 0.891 | 0.99  (0.87, 1.14) | 0.937 |
| Other | 1.10  (0.75, 1.61) | 0.637 | 1.07  (0.75, 1.53) | 0.700 | 1.07  (0.75, 1.51) | 0.715 |

| S3 Table (continued). | | | | | | |
| --- | --- | --- | --- | --- | --- | --- |
| **Characteristic** | **Model 1** | | **Model 2** | | **Model 3** | |
|  | **aPR**  **(95% CI)** | **p-value** | **aPR**  **(95% CI)** | **p-value** | **aPR**  **(95% CI)** | **p-value** |
| **Shared toilet** |  |  |  |  |  |  |
| No | 1.0 | - | 1.0 | - | 1.0 | - |
| Yes | 1.09  (1.00, 1.20) | 0.056 | 1.10  (0.98, 1.23) | 0.110 | 1.09  (0.99, 1.21) | 0.093 |
| **Electricity** |  |  |  |  |  |  |
| Yes | 1.0 | - | 1.0 | - | 1.0 | - |
| No | 1.06  (0.88, 1.28) | 0.552 | 1.06  (0.89, 1.26) | 0.515 | 1.04  (0.87, 1.24) | 0.667 |
| **Refrigerator** |  |  |  |  |  |  |
| Yes | 1.0 | - | 1.0 | - | 1.0 | - |
| No | 1.50  (1.21, 1.85) | <0.001 | 1.48  (1.19, 1.84) | <0.001 | 1.50  (1.20, 1.86) | <0.001 |
| **Type of flooring** |  |  |  |  |  |  |
| Earth/ sand/ gravel | 1.07  (0.94, 1.23) | 0.306 | 1.06  (0.91, 1.23) | 0.449 | 1.05  (0.90, 1.22) | 0.514 |
| Cement | 1.0 | - | 1.0 | - | 1.0 | - |
| Ceramic/ stone | 1.04  (0.84, 1.28) | 0.737 | 1.03  (0.84, 1.27) | 0.745 | 1.09  (0.87, 1.38) | 0.450 |
| Other | 1.77  (1.36, 2.29) | <0.001 | 1.74  (1.33, 2.29) | <0.001 | 1.81  (1.35, 2.42) | <0.001 |
| **Cooking fuel** |  |  |  |  |  |  |
| Gas | 1.0 | - | 1.0 | - | 1.0 | - |
| Charcoal | 0.91  (0.80, 1.03) | 0.131 | 0.91  (0.80, 1.03) | 0.132 | 0.88  (0.79, 0.99) | 0.028 |
| Biomass | 0.98  (0.87, 1.11) | 0.770 | 0.98  (0.86, 1.11) | 0.753 | 0.95  (0.83, 1.09) | 0.443 |
| Other | 1.23  (0.96, 1.58) | 0.101 | 1.23  (0.98, 1.53) | 0.076 | 1.19  (0.93, 1.53) | 0.158 |
| **Household size, residents** |  |  |  |  |  |  |
| One to four | 1.0 | - | 1.0 | - | 1.0 | - |
| Five to six | 1.02  (0.90, 1.14) | 0.805 | 1.01  (0.90, 1.14) | 0.831 | 1.01  (0.89, 1.15) | 0.830 |
| Seven or more | 0.94  (0.81, 1.09) | 0.415 | 0.94  (0.80, 1.09) | 0.407 | 0.95  (0.81, 1.12) | 0.520 |
| **Eligible women in household** |  |  |  |  |  |  |
| One | 1.0 | - | 1.0 | - | 1.0 | - |
| Two | 1.06  (0.92, 1.22) | 0.427 | 1.05  (0.91, 1.22) | 0.479 | 1.05  (0.91, 1.21) | 0.530 |
| Three or more | 1.07  (0.88, 1.30) | 0.486 | 1.08  (0.89, 1.30) | 0.453 | 1.05  (0.89, 1.25) | 0.555 |
| S3 Table (continued). | | | | | | |
| **Characteristic** | **Model 1** | | **Model 2** | | **Model 3** | |
|  | **aPR**  **(95% CI)** | **p-value** | **aPR**  **(95% CI)** | **p-value** | **aPR**  **(95% CI)** | **p-value** |
| **Eligible children in household** |  |  |  |  |  |  |
| One | 0.87  (0.79, 0.97) | 0.010 | 0.87  (0.77, 0.98) | 0.019 | 0.89  (0.79, 1.00) | 0.052 |
| Two | 1.0 | - | 1.0 | - | 1.0 | - |
| Three or more | 0.96  (0.83, 1.11) | 0.605 | 0.97  (0.84, 1.11) | 0.633 | 0.96  (0.82, 1.12) | 0.579 |
| **Wealth Index** |  |  |  |  |  |  |
| Poorest | 0.98  (0.81, 1.18) | 0.797 | 0.98  (0.83, 1.16) | 0.792 | 1.00  (0.84, 1.19) | 0.994 |
| Poorer | 0.94  (0.79, 1.13) | 0.522 | 0.94  (0.81, 1.10) | 0.471 | 0.96  (0.82, 1.13) | 0.634 |
| Middle | 1.0 | - | 1.0 | - | 1.0 | - |
| Wealthier | 1.10  (0.95, 1.28) | 0.200 | 1.09  (0.95, 1.26) | 0.211 | 1.08  (0.94, 1.25) | 0.277 |
| Wealthiest | 1.16  (0.87, 1.56) | 0.316 | 1.17  (0.85, 1.60) | 0.326 | 1.15  (0.88, 1.50) | 0.311 |
| **Area of residence** |  |  |  |  |  |  |
| Urban | 1.0 | - | 1.0 | - | 1.0 | - |
| Rural | 1.00  (0.90, 1.12) | 0.941 | 1.00  (0.89, 1.12) | 0.982 | 0.97  (0.87, 1.07) | 0.530 |
| Numbers reported are prevalence ratio adjusted for confounding and clustering of observations using multivariable, multilevel, mixed-effects, Poisson regression (aPR), 95% confidence interval (95% CI), and p-value of the hypothesis test that prevalence of each level of exposure equals the prevalence for the baseline level of variable. The three levels of multilevel mixed-effects model were: individual children nested within PSUs, nested within provinces. | | | | | | |
