# Supplementary material for: Factors associated with stunting among children 0 to 59 months of age in Angola: A cross-sectional study using the 2015–2016 Demographic and Health Survey
Source: PLOS Glob Public Health. 2022 Dec 12;2(12):e0000983. doi: 10.1371/journal.pgph.0000983 (PMC10021435; doi:10.1371/journal.pgph.0000983)
Supplement: S4 Table — Value reported are crude absolute risk difference (ARD) and absolute risk difference adjusted for confounding using multivariable, fixed-effects, Poisson regression (aARD), 95% confidence interval (95% CI), and p-value of the hypothesis test that prevalence of each level of exposure equals the prevalence for the baseline level of variable. (DOCX) [file pgph.0000983.s005.docx]

**S4 Table. Crude and adjusted absolute risk difference (ARD) of stunting according to characteristics of participants and households.**

| **Characteristic** | **Crude** | | **Model 1** | | **Model 2** | | **Model 3** | | |
| --- | --- | --- | --- | --- | --- | --- | --- | --- | --- |
|  | **ARD, % (95% CI)** | **p-value** | **aARD, % (95%CI)** | **p-value** | **aARD, % (95%CI)** | **p-value** | **aARD, % (95%CI)** | **p-value** |  |
| **Sex of child** |  |  |  |  |  |  |  |  |  |
| Female | reference | - | reference | - | reference | - | reference | - |  |
| Male | 7.6 (4.6, 10.6) | <0.001 | 7.0 (4.3, 9.7) | <0.001 | 7.0 (4.2, 9.7) | <0.001 | 6.9 (4.1, 9.6) | <0.001 |  |
| **Child age, months** |  |  |  |  |  |  |  |  |  |
| 0 to 11 | reference | - | reference | - | reference | - | reference | - |  |
| 12 to 23 | 24.9 (19.9, 29.9) | <0.001 | 23.7 (19.2, 28.2) | <0.001 | 23.6 (19.1, 28.2) | <0.001 | 23.1 (18.6, 27.6) | <0.001 |  |
| 24 to 35 | 28.0 (23.2, 32.8) | <0.001 | 29.8 (24.8, 34.8) | <0.001 | 29.8 (23.1, 36.4) | <0.001 | 29.4 (22.8, 36.0) | <0.001 |  |
| 36 to 47 | 21.2 (16.4, 26.0) | <0.001 | 23.1 (18.4, 27.9) | <0.001 | 23.4 (17.2, 29.7) | <0.001 | 24.0 (17.7, 30.3) | <0.001 |  |
| 48 to 59 | 12.3 (7.6, 17.1) | <0.001 | 13.2 (8.4, 18.0) | <0.001 | 13.4 (7.2, 19.6) | <0.001 | 13.7 (7.5, 20.0) | <0.001 |  |
| **Birth order** |  |  |  |  |  |  |  |  |  |
| First | reference | - | reference | - | reference | - | reference | - |  |
| Second | 0.3 (-4.5, 5.1) | 0.898 | 0.6 (-4.2, 5.4) | 0.795 | 1.0 (-3.9, 6.00) | 0.682 | 1.4 (-3.5, 6.3) | 0.565 |  |
| Third and fourth | 4.5 (-1.0, 9.9) | 0.108 | 7.3 (1.8, 12.8) | 0.009 | 7.7 (1.9, 13.6) | 0.010 | 7.9 (2.1, 13.7) | 0.008 |  |
| Fifth and above | 5.1 (0.4, 9.8) | 0.034 | 10.8 (3.4, 18.1) | 0.004 | 11.5 (3.6, 19.3) | 0.004 | 11.6 (3.8, 19.4) | 0.004 |  |
| **Birthweight (grams)** |  |  |  |  |  |  |  |  |  |
| Low (< 2,500) | 14.4 (6.6, 22.1) | <0.001 | - | - | - | - | 12.7 (4.6, 20.7) | 0.002 |  |
| Normal (2,500 to 3,999) | reference | - | - | - | - | - | reference | - |  |
| High (≥ 4,000) | -4.2 (-9.7, 1.4) | 0.140 | - | - | - | - | -8.7 (-13.7, -3.7) | 0.001 |  |
| Not weighed at birth | 16.9 (12.5, 21.2) | <0.001 | - | - | - | - | 2.8 (-1.1, 6.7) | 0.162 |  |
| Missing | 14.0 (4.8, 23.1) | 0.003 | - | - | - | - | 4.8 (-2.6, 12.3) | 0.204 |  |

| S4 Table (continued) | | | | | | | | |
| --- | --- | --- | --- | --- | --- | --- | --- | --- |
| **Characteristic** | **Crude** |  | Model 1 |  | Model 2 |  | Model 3 | |
|  | **ARD, % (95% CI)** | **p-value** | **aARD, %(95% CI)** | **p-value** | **aARD, % (95% CI)** | **p-value** | **aARD, % (95% CI)** | **p-value** |
| **Diarrhea in last 2 weeks** |  |  |  |  |  |  |  |  |
| No | reference | - | - | - | - | - | reference | - |
| Yes | 10.0 (5.3, 14.8) | <0.001 | - | - | - | - | 8.5 (3.9, 13.1) | <0.001 |
| Missing | -16.7 (-33.4, 0.1) | 0.051 | - | - | - | - | -27.0 (-36.8, -17.1) | <0.001 |
| **Fever in last 2 weeks** |  |  |  |  |  |  |  |  |
| No | reference | - | - | - | - | - | reference | - |
| Yes | 3.3 (-1.6, 8.2) | 0.189 | - | - | - |  | -0.6 (-4.9, 3.8) | 0.804 |
| Missing | -1.3 (-23.3, 20.7) | 0.907 | - | - | - | - | - | - |
| **Cough in last 2 weeks** |  |  |  |  |  |  |  |  |
| No | reference | - | - | - | - | - | reference | - |
| Yes | 0.0 (-5.2, 5.2) | 0.993 | - | - | - |  | -0.8 (-5.1, 3.5) | 0.730 |
| Missing | 1.6 (-29.6, 29.9) | 0.991 | - | - | - | - | -9.6 (-54.6, 35.4) | 0.676 |
| **Maternal age, years** |  |  |  |  |  |  |  |  |
| 15 to 19 | 0.1 (-6.3, 6.5) | 0.975 | 10.7 (2.0, 19.4) | 0.016 | 13.3 (3.5, 23.1) | 0.008 | 12.5 (2.6, 22.3) | 0.013 |
| 20 to 24 | 4.2 (-0.7, 9.1) | 0.093 | 9.2 (2.5, 15.9) | 0.007 | 10.5 (3.6, 17.3) | 0.003 | 9.0 (2.2, 15.8) | 0.010 |
| 25 to 29 | -1.7 (-7.1, 3.6) | 0.527 | 2.8 (-2.6, 8.2) | 0.313 | 3.8 (-1.6, 9.1) | 0.165 | 3.3 (-2.1, 8.6) | 0.232 |
| 30 to 34 | -0.9 (-7.1, 5.4) | 0.781 | 2.5 (-2.3, 7.2) | 0.307 | 2.7 (-2.0, 7.4) | 0.256 | 3.1 (-1.8, 8.1) | 0.219 |
| 35 and older | reference | - | reference | - | reference | - | reference | - |
| **Maternal education,** |  |  |  |  |  |  |  |  |
| No formal education | reference | - | reference | - | reference | - | reference | - |
| Primary | -6.2 (-10.2, -2.1) | 0.003 | -1.6 (-5.5, 2.3) | 0.430 | -1.4 (-5.3, 2.6) | 0.500 | -0.7 (-4.7, 3.3) | 0.735 |
| Secondary | -20.7 (-25.1, -16.4) | <0.001 | -9.1 (-14.8, -3.4) | 0.002 | -8.8 (-14.6, -3.0) | 0.003 | -7.9 (-14.0, -1.9) | 0.010 |
| Higher | -37.5 (-44.8, -30.3) | <0.001 | -26.1 (-36.0, -16.2) | <0.001 | -26.6 (-35.8, -17.4) | <0.001 | -25.7 (-35.4, -16.0) | <0.001 |

| S4 Table (continued) | | | | | | | | |
| --- | --- | --- | --- | --- | --- | --- | --- | --- |
| **Characteristic** | **Crude** | | **Model 1** | | **Model 2** | | **Model 3** | |
|  | **ARD, % (95% CI)** | **p-value** | **aARD, % (95% CI)** | **p-value** | **aARD, % (95% CI)** | **p-value** | **aARD, % (95% CI)** | **p-value** |
| **Cohabitation status** |  |  |  |  |  |  |  |  |
| Living together | reference | - | reference | - | reference | - | reference | - |
| Living separated | 4.4 (-1.4, 10.2) | 0.135 | 1.7 (-3.1, 6.5) | 0.484 | 2.0 (-2.9, 6.9) | 0.430 | 1.8 (-3.1, 6.8) | 0.463 |
| Widowed, divorced | 7.1 (0.2, 14.0) | 0.044 | 3.1 (-2.4, 8.7) | 0.271 | 3.1 (-2.4, 8.7) | 0.272 | 3.3 (-2.4, 8.9) | 0.256 |
| Never in a union | -1.6 (-6.8, 3.6) | 0.545 | 2.1 (-2.8, 7.0) | 0.394 | 2.2 (-2.7, 7.2) | 0.383 | 2.5 (-2.6, 7.6) | 0.329 |
| **Age of sexual initiation, years** |  |  |  |  |  |  |  |  |
| 14 and younger | 5.3 (0.7, 9.9) | 0.023 | - | - | -3.0 (-7.5, 1.5) | 0.190 | -2.6 (-7.1, 1.9) | 0.256 |
| 15 to 16 | 5.4 (1.3, 9.6) | 0.011 | - | - | -0.8 (-5.0, 3.4) | 0.720 | -0.8 (-5.0, 3.4) | 0.711 |
| 17 and older | reference | - | - | - | reference | - | reference | - |
| **Sexual autonomy** |  |  |  |  |  |  |  |  |
| Yes | reference | - | - | - | reference | - | reference | - |
| No | 8.1 (3.6, 12.5) | <0.001 | - | - | 0.00 (-4.5, 4.6) | 0.988 | 0.1 (-4.6, 4.5) | 0.979 |
| **Safe sex autonomy** |  |  | - |  |  |  |  |  |
| Yes | reference | - | - | - | reference | - | reference | - |
| No | 11.2 (6.7, 15.7) | <0.001 | - | - | 1.2 (-3.4, 5.7) | 0.611 | 0.8 (-3.7, 5.3) | 0.712 |
| **Lifetime natality control** |  |  |  |  |  |  |  |  |
| Yes | reference | - | - | - | reference | - | reference | - |
| No | 11.9 (6.9, 16.9) | <0.001 | - | - | 1.3 (-4.2, 6.8) | 0.637 | 1.7 (-3.8, 7.1) | 0.547 |
| **Antenatal care, visits** |  |  |  |  |  |  |  |  |
| < 4 visits | 12.5 (7.8, 17.1) | <0.001 | - | - | 3.9 (-0.4, 8.3) | 0.076 | 3.3 (-1.1, 7.8) | 0.139 |
| ≥ 4 visits | reference | - | - | - | reference | - | reference | - |
| Missing | 13.1 (8.6, 17.6) | <0.001 | - | - | 7.9 (-9.0, 24.7) | 0.360 | 6.1 (-11.0, 23.2) | 0.482 |

| S4 Table (continued) | | | | | | | | |
| --- | --- | --- | --- | --- | --- | --- | --- | --- |
| **Characteristic** | **Crude** | | **Model 1** | | **Model 2** | | **Model 3** | |
|  | **ARD, % (95% CI)** | **p-value** | **aARD, % (95% CI)** | **p-value** | **aARD, % (95% CI)** | **p-value** | **aARD, % (95% CI)** | **p-value** |
| **Newborn health visit** |  |  |  |  |  |  |  |  |
| No | reference | - | - | - | reference | - | reference | - |
| Yes | -1.6 (-6.4, 3.1) | 0.498 | - | - | 2.1 (-3.5, 7.8) | 0.454 | 0.4 (-5.0, 5.8) | 0.889 |
| Missing | 7.9 (4.1, 11.7) | <0.001 | - | - | -6.6 (-22.6, 9.3) | 0.415 | -5.1 (-21.6, 11.3) | 0.541 |
| **Breastfeeding** |  |  |  |  |  |  |  |  |
| Currently | reference | - | - | - | reference | - | reference | - |
| Not currently | 10.0 (6.6, 13.3) | <0.001 | - | - | 0.7 (-4.1, 5.5) | 0.774 | 1.0 (-3.9, 5.8) | 0.696 |
| Never breastfed | 14.8 (4.8, 24.7) | 0.004 | - | - | 3.5 (-5.1, 12.1) | 0.426 | 3.1 (-5.5, 11.8) | 0.478 |
| **Healthcare decision** |  |  |  |  |  |  |  |  |
| Joint | reference | - | - | - | reference | - | reference | - |
| Husband/ partner/ other | 1.6 (-2.7, 5.9) | 0.470 | - | - | -0.3 (-4.4, 3.9) | 0.903 | -0.3 (-4.4, 3.8) | 0.890 |
| Woman | 3.4 (-1.4, 8.3) | 0.167 | - | - | 4.7 (-0.4, 9.8) | 0.074 | 4.5 (-0.7, 9.6) | 0.092 |
| **Decision to visit family** |  |  |  |  |  |  |  |  |
| Joint | reference | - | - | - | reference | - | reference | - |
| Husband/ partner/ other | 2.3 (-3.3, 7.9) | 0.416 | - | - | 0.4 (-5.3, 6.1) | 0.884 | 0.1 (-5.5, 5.7) | 0.970 |
| Woman | 4.3 (-0.1, 8.8) | 0.059 | - | - | 2.2 (-2.3, 6.7) | 0.343 | 1.8 (-2.7, 6.4) | 0.423 |
| **Work outside of home** |  |  |  |  |  |  |  |  |
| Not working | -7.3 (-12.0, -2.7) | 0.002 | - | - | -2.3 (-7.3, 2.7) | 0.364 | -2.2 (-7.3, 2.8) | 0.392 |
| Working | reference | - | - | - | reference | - | reference | - |

| S4 Table (continued) | | | | | | | | | |
| --- | --- | --- | --- | --- | --- | --- | --- | --- | --- |
| **Characteristic** | **Crude** | | **Model 1** | | **Model 2** | | **Model 3** | | |
|  | **ARD, % (95%CI)** | **p-value** | **aARD, % (95%CI)** | **p-value** | **aARD, % (95%CI)** | **p-value** | **aARD, % (95%CI)** | **p-value** |  |
| **Paternal age, years** |  |  |  |  |  |  |  |  |  |
| 15 to 19 | -1.7 (-15.7, 12.3) | 0.816 | 1.7 (-14.8, 18.1) | 0.843 | 1.1 (-15.3, 17.5) | 0.895 | 1.0 (-15.4, 17.4) | 0.909 |  |
| 20 to 24 | 9.1 (2.8, 15.4) | 0.005 | 8.6 (0.4, 16.9) | 0.041 | 8.6 (0.2, 17.1) | 0.044 | 8.4 (0.00, 16.8) | 0.049 |  |
| 25 to 29 | 4.5 (-0.8, 9.9) | 0.095 | 3.2 (-2.8, 9.2) | 0.296 | 3.2 (-2.9, 9.2) | 0.305 | 3.3 (-2.8, 9.3) | 0.291 |  |
| 30 to 34 | 2.6 (-3.7, 9.0) | 0.416 | 2.2 (-3.8, 8.1) | 0.476 | 2.2 (-3.8, 8.2) | 0.473 | 2.6 (-3.2, 8.5) | 0.376 |  |
| 35 and older | reference | - | reference | - | reference | - | reference | - |  |
| Missing | -0.7 (-10.0, 8.5) | 0.875 | -4.7 (-12.2, 2.8) | 0.222 | -4.8 (-12.3, 2.7) | 0.209 | -4.9 (-12.3, 2.5) | 0.194 |  |
| **Paternal education,** |  |  |  |  |  |  |  |  |  |
| No formal education | reference | - | reference | - | reference | - | reference | - |  |
| Primary | -3.4 (-9.1, 2.3) | 0.237 | 0.00 (-4.8, 4.8) | 0.996 | -0.2 (-5.1, 4.6) | 0.926 | -0.8 (-5.8, 4.1) | 0.745 |  |
| Secondary | -16.2 (-22.3, -10.2) | <0.001 | -3.2 (-9.0, 2.7) | 0.287 | -3.0 (-8.8, 2.7) | 0.301 | -3.3 (-9.1, 2.5) | 0.263 |  |
| Higher | -34.1 (-41.5, -26.6) | <0.001 | -14.0 (-25.7, -2.2) | 0.020 | -13.8 (-25.6, -1.9) | 0.023 | -14.5 (-26.2, -2.8) | 0.015 |  |
| Missing | -10.3 (-18.9, -1.7) | 0.019 | -3.3 (-10.1, 3.5) | 0.337 | -3.5 (-10.3, 3.4) | 0.319 | -3.3 (-10.3, 3.8) | 0.365 |  |
| **Source of drinking water** |  |  |  |  |  |  |  |  |  |
| Piped | reference | - | reference | - | reference | - | reference | - |  |
| Fountain, well | 15.7 (9.5, 21.9) | <0.001 | 9.7 (3.3, 16.1) | 0.003 | 9.6 (3.3, 15.9) | 0.003 | 9.4 (3.1, 15.7) | 0.003 |  |
| Spring/ surface/ rain | 21.3 (15.3, 27.2) | <0.001 | 10.0 (3.1, 16.9) | 0.004 | 9.8 (2.9, 16.7) | 0.005 | 9.7 (2.8, 16.7) | 0.006 |  |
| Other | 4.5 (-2.3, 11.2) | 0.197 | 2.6 (-3.8, 9.1) | 0.420 | 2.4 (-4.0, 8.9) | 0.461 | 2.8 (-3.8, 9.4) | 0.413 |  |

| S4 Table (continued) | | | | | | | | | | |
| --- | --- | --- | --- | --- | --- | --- | --- | --- | --- | --- |
| **Characteristic** | **Crude** | | | **Model 1** | | **Model 2** | | **Model 3** | | |
|  | **ARD, % (95% CI)** | **p-value** | **aARD, % (95%CI)** | | **p-value** | **aARD, % (95%CI)** | **p-value** | **aARD, % (95%CI)** | **p-value** |  |
| **Sanitary system** |  |  |  | |  |  |  |  |  |  |
| Septic tank | reference | - | reference | | - | reference | - | reference | - |  |
| Public sanitary sewer | 2.2 (-6.9, 11.3) | 0.635 | 5.7 (-3.2, 14.5) | | 0.207 | 6.4 (-2.6, 15.3) | 0.162 | 8.0 (-1.5, 17.5) | 0.099 |  |
| Open pit | 18.8 (12.3, 25.3) | <0.001 | 8.5 (2.5, 14.5) | | 0.006 | 8.4 (2.4, 14.4) | 0.006 | 8.0 (1.9, 14.1) | 0.010 |  |
| No sanitary facility | 12.9 (8.9, 16.9) | <0.001 | -0.1 (-4.8, 4.6) | | 0.977 | -0.3 (-5.0, 4.4) | 0.903 | -0.5 (-5.2, 4.2) | 0.837 |  |
| Other | 9.8 (-8.0, 27.5) | 0.280 | 6.7 (-7.5, 20.8) | | 0.358 | 4.5 (-9.5, 18.5) | 0.527 | 4.6 (-9.7, 18.8) | 0.530 |  |
| **Shared toilet** |  |  |  | |  |  |  |  |  |  |
| No | reference | - | reference | | - | reference | - | reference | - |  |
| Yes | 6.9 (1.5, 12.3) | 0.013 | 4.3 (-0.6, 9.1) | | 0.088 | 4.3 (-0.4, 9.1) | 0.076 | 4.1 (-0.8, 9.0) | 0.099 |  |
| **Electricity** |  |  |  | |  |  |  |  |  |  |
| Yes | reference | - | reference | | - | reference | - | reference | - |  |
| No | 16.8 (12.4, 21.2) | <0.001 | 2.4 (-3.8, 8.5) | | 0.451 | 2.3 (-3.7, 8.4) | 0.454 | 1.5 (-4.6, 7.6) | 0.637 |  |
| **Refrigerator** |  |  |  | |  |  |  |  |  |  |
| Yes | reference | - | reference | | - | reference | - | reference | - |  |
| No | 21.1 (16.5, 25.7) | <0.001 | 14.0 (7.1, 20.9) | | <0.001 | 13.6 (7.0, 20.3) | <0.001 | 13.8 (7.0, 20.6) | <0.001 |  |
| **Type of flooring** |  |  |  | |  |  |  |  |  |  |
| Earth/ sand/ gravel | 15.4 (10.8, 19.9) | <0.001 | 2.5 (-4.0, 9.0) | | 0.452 | 1.9 (-4.5, 8.4) | 0.558 | 1.6 (-4.8, 8.0) | 0.626 |  |
| Cement | reference | - | reference | | - | reference | - | reference | - |  |
| Ceramic/ stone | -8.8 (-15.4, -2.2) | 0.009 | 0.3 (-10.1, 10.7) | | 0.958 | 0.3 (-10.0, 10.6) | 0.951 | 2.5 (-8.9, 13.8) | 0.672 |  |
| Other | 29.9 (8.7, 51.1) | 0.006 | 25.8 (5.8, 45.7) | | 0.011 | 25.9 (5.5, 46.2) | 0.013 | 27.1 (4.9, 49.2) | 0.017 |  |

| S4 Table (continued) | | | | | | | | | | | |  |  |
| --- | --- | --- | --- | --- | --- | --- | --- | --- | --- | --- | --- | --- | --- |
| **Characteristic** | **Crude** | | **Model 1** | | | **Model 2** | | | **Model 3** | | | |  |
|  | **ARD, % (95% CI)** | **p-value** | | **aARD, % (95%CI)** | **p-value** | | **aARD, % (95%CI)** | **p-value** | | **aARD, % (95%CI)** | **p-value** | | |
| **Cooking fuel** |  |  | |  |  | |  |  | |  |  | | |
| Gas | reference | - | | reference | - | | reference | - | | reference | - | | |
| Charcoal | 10.4 (5.2, 15.6) | <0.001 | | -1.5 (-7.5, 4.4) | 0.619 | | -2.0 (-7.9, 3.8) | 0.497 | | -3.2 (-8.7, 2.4) | 0.269 | | |
| Biomass | 18.1 (13.4, 22.8) | <0.001 | | 1.6 (-4.9, 8.1) | 0.622 | | 1.0 (-5.4, 7.5) | 0.744 | | -0.1 (-6.3, 6.1) | 0.978 | | |
| Other | 15.2 (5.5, 24.8) | 0.002 | | 11.6 (1.5, 21.8) | 0.024 | | 10.9 (1.1, 20.7) | 0.030 | | 9.6 (-0.8, 20.0) | 0.071 | | |
| **Household size, residents** |  |  | |  |  | |  |  | |  |  | | |
| One to four | reference | - | | reference | - | | reference | - | | reference | - | | |
| Five to six | 2.6 (-2.5, 7.6) | 0.315 | | 0.4 (-4.3, 5.1) | 0.863 | | 0.4 (-4.3, 5.0) | 0.876 | | 0.3 (-4.3, 4.9) | 0.901 | | |
| Seven or more | -2.2 (-6.6, 2.3) | 0.348 | | -2.6 (-8.4, 3.2) | 0.382 | | -2.5 (-8.4, 3.3) | 0.400 | | -2.1 (-8.0, 3.7) | 0.473 | | |
| **Eligible women in household** |  |  | |  |  | |  |  | |  |  | | |
| One | reference | - | | reference | - | | reference | - | | reference | - | | |
| Two | -4.0 (-9.0, 1.1) | 0.122 | | 2.3 (-3.4, 8.1) | 0.429 | | 2.0 (-3.6, 7.7) | 0.480 | | 1.8 (-3.8, 7.3) | 0.532 | | |
| Three or more | -8.1 (-15.1, -1.0) | 0.024 | | 3.8 (-5.6, 13.2) | 0.428 | | 3.7 (-5.6, 13.0) | 0.436 | | 2.7 (-6.3, 11.7) | 0.552 | | |
| **Eligible children in household** |  |  | |  |  | |  |  | |  |  | | |
| One | -9.7 (-13.7, -5.7) | <0.001 | | -5.0 (-8.9, -1.1) | 0.012 | | -5.3 (-9.5, -1.0) | 0.016 | | -4.5 (-8.8, -0.3) | 0.036 | | |
| Two | reference | - | | reference | - | | reference | - | | reference | - | | |
| Three or more | -0.4 (-4.6, 3.8) | 0.850 | | -1.6 (-5.2, 2.0) | 0.398 | | -1.4 (-4.9, 2.2) | 0.453 | | -1.6 (-5.0, 2.0) | 0.383 | | |
| **Wealth Index** |  |  | |  |  | |  |  | |  |  | | |
| Poorest | 9.3 (3.9, 14.7) | 0.001 | | -2.8 (-11.0, 5.5) | 0.507 | | -2.3 (-10.5, 5.8) | 0.570 | | -1.2 (-9.3, 7.0) | 0.779 | | |
| Poorer | 6.8 (1.7, 11.9) | 0.009 | | -4.6 (-11.6, 2.3) | 0.191 | | -4.0 (-10.7, 2.7) | 0.245 | | -3.1 (-9.8, 3.5) | 0.353 | | |
| Middle | reference | - | | reference | - | | reference | - | | reference | - | | |
| Wealthier | -10.6 (-17.7, -3.5) | 0.003 | | 3.1 (-6.3, 12.6) | 0.516 | | 3.0 (-6.5, 12.5) | 0.535 | | 2.1 (-7.1, 11.2) | 0.658 | | |
| Wealthiest | -18.2 (-24.5, -11.8) | <0.001 | | 6.2 (-10.7, 23.1) | 0.471 | | 6.9 (-10.0, 23.8) | 0.422 | | 5.2 (-10.9, 21.3) | 0.528 | | |

| S4 Table (continued) | | | | | | | | | | | | | |  |
| --- | --- | --- | --- | --- | --- | --- | --- | --- | --- | --- | --- | --- | --- | --- |
| **Characteristic** | **Crude** | | **Model 1** | | **Model 2** | | | **Model 3** | | | | | | |
|  | **ARD, % (95% CI)** | **p-value** | **aARD, % (95%CI)** | **p-value** | **aARD, % (95%CI)** | **p-value** | | **aARD, % (95%CI)** | | **p-value** | | | | |
| **Area of residence** |  |  |  |  |  | |  | |  | |  | |  |  |
| Urban | reference | - | reference | - | reference | | - | | reference | | - | |  |  |
| Rural | 14.2 (10.0, 18.4) | <0.001 | 0.3 (-4.6, 5.1) | 0.912 | 0.1 (-4.7, 4.8) | | 0.968 | | -0.9 (-5.6, 3.7) | | 0.692 | |  |  |
| **Provinces** |  |  |  |  |  | |  | |  | |  | |  |  |
| Bengo | 9.5 (1.3, 17.8) | 0.024 | -5.2 (-14.9, 4.5) | 0.295 | -7.0 (-16.8, 2.9) | | 0.165 | | -4.9 (-15.1, 5.3) | | 0.344 | |  |  |
| Benguela | 2.4 (-5.5, 10.3) | 0.553 | -12.5 (-21.4, -3.6) | 0.006 | -14.0 (-22.7, -5.1) | | 0.002 | | -13.3 (-21.9, -4.8) | | 0.002 | |  |  |
| Bié | 19.7 (10.9, 28.4) | <0.001 | -5.2 (-14.5, 4.2) | 0.280 | -5.7 (-15.0, 3.6) | | 0.227 | | -4.6 (-14.0, 4.9) | | 0.341 | |  |  |
| Cabinda | -7.9 (-16.8, 1.0) | 0.083 | -20.0 (-29.3, -10.7) | <0.001 | -20.0 (-29.4, -10.5) | | <0.001 | | -19.0 (-28.3, -9.7) | | <0.001 | |  |  |
| Cuando-Cubango | 14.9 (4.1, 25.7) | 0.007 | -4.2 (-15.4, 7.1) | 0.469 | -4.2 (-15.6, 7.3) | | 0.473 | | -4.1 (-15.4, 7.3) | | 0.485 | |  |  |
| Cuanza Norte | 16.8 (7.2, 26.4) | 0.001 | -1.5 (-11.7, 8.7) | 0.769 | -1.8 (-11.9, 8.3) | | 0.723 | | -1.7 (-11.8, 8.3) | | 0.733 | |  |  |
| Cuanza Sul | 18.1 (9.5, 26.6) | <0.001 | -5.6 (-14.8, 3.6) | 0.232 | -6.7 (-15.8, 2.4) | | 0.148 | | -6.0 (-14.9, 2.8) | | 0.181 | |  |  |
| Cunene | 5.5 (-2.6, 13.6) | 0.180 | -12.0 (-21.4, -2.6) | 0.012 | -11.8 (-21.3, -2.4) | | 0.014 | | -10.3 (-19.9, -0.8) | | 0.034 | |  |  |
| Huíla | 12.4 (3.6, 21.3) | 0.006 | -8.6 (-18.0, 0.9) | 0.075 | -9.1 (-18.5, 0.3) | | 0.058 | | -8.4 (-17.8, 1.0) | | 0.080 | |  |  |
| Huambo | 13.6 (3.1, 24.2) | 0.012 | -7.3 (-17.6, 3.1) | 0.168 | -7.6 (-17.7, 2.5) | | 0.142 | | -6.8 (-16.8, 3.2) | | 0.184 | |  |  |
| Luanda | reference | - | reference | - | reference | | - | | reference | | - | |  |  |
| Lunda Norte | 8.2 (-1.9, 18.4) | 0.111 | -7.5 (-18.1, 3.2) | 0.168 | -7.8 (-18.6, 3.0) | | 0.157 | | -5.8 (-16.9, 5.4) | | 0.311 | |  |  |
| Lunda Sul | 13.6 (5.3, 22.0) | 0.001 | -0.8 (-10.1, 8.6) | 0.874 | -1.1 (-11.0, 8.8) | | 0.829 | | 2.0 (-8.2, 12.2) | | 0.699 | |  |  |
| Malanje | 4.5 (-4.3, 13.3) | 0.314 | -12.2 (-22.0, -2.4) | 0.015 | -12.8 (-22.8, -2.9) | | 0.011 | | -12.2 (-21.8, -2.5) | | 0.013 | |  |  |
| Moxico | 8.7 (-1.8, 19.2) | 0.104 | -11.7 (-22.0, -1.5) | 0.024 | -11.7 (-22.1, -1.4) | | 0.026 | | -10.9 (-21.2, -0.6) | | 0.037 | |  |  |
| Namibe | 5.2 (-3.2, 13.6) | 0.222 | -6.5 (-16.4, 3.4) | 0.197 | -7.3 (-17.2, 2.6) | | 0.149 | | -6.3 (-16.1, 3.5) | | 0.206 | |  |  |
| Uíge | 11.3 (3.0, 19.6) | 0.007 | -6.6 (-16.4, 3.1) | 0.182 | -7.5 (-17.2, 2.3) | | 0.135 | | -6.0 (-15.6, 3.7) | | 0.225 | |  |  |
| Zaire | -5.4 (-13.9, 3.0) | 0.208 | -19.9 (-29.6, -10.3) | <0.001 | -18.8 (-28.6, -9.1) | | <0.001 | | -17.2 (-26.8, -7.7) | | <0.001 | |  |  |
| Numbers reported are crude absolute risk difference (ARD) and absolute risk difference adjusted for confounding using multivariable, fixed-effects, Poisson regression (aARD), 95% confidence interval (95% CI), and p-value of the hypothesis test that prevalence of each level of exposure equals the prevalence for the baseline level of variable. | | | | | | | | | | | |  |  |  |
