# Supplementary figures and images for: Factors associated with stunting among children 0 to 59 months of age in Angola: A cross-sectional study using the 2015–2016 Demographic and Health Survey
Source: PLOS Glob Public Health. 2022 Dec 12;2(12):e0000983. doi: 10.1371/journal.pgph.0000983 (PMC10021435; doi:10.1371/journal.pgph.0000983)

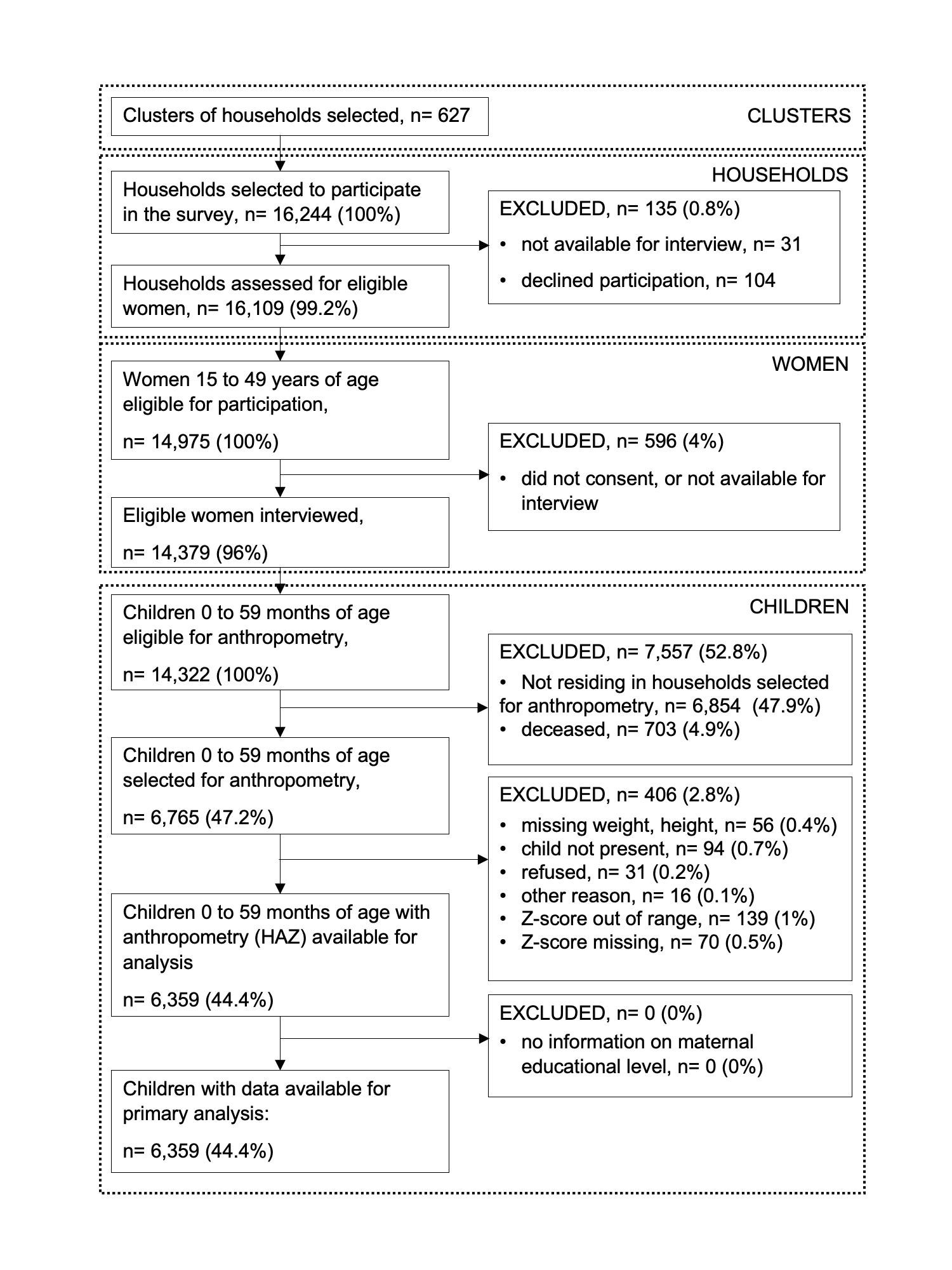

Supplement: S1 Fig — Flowchart describing number of clusters (primary sampling units), households, women, and children selected for participation, number excluded with reason for exclusion, and number of participants included in study sample. (TIF) [file pgph.0000983.s006.tif]

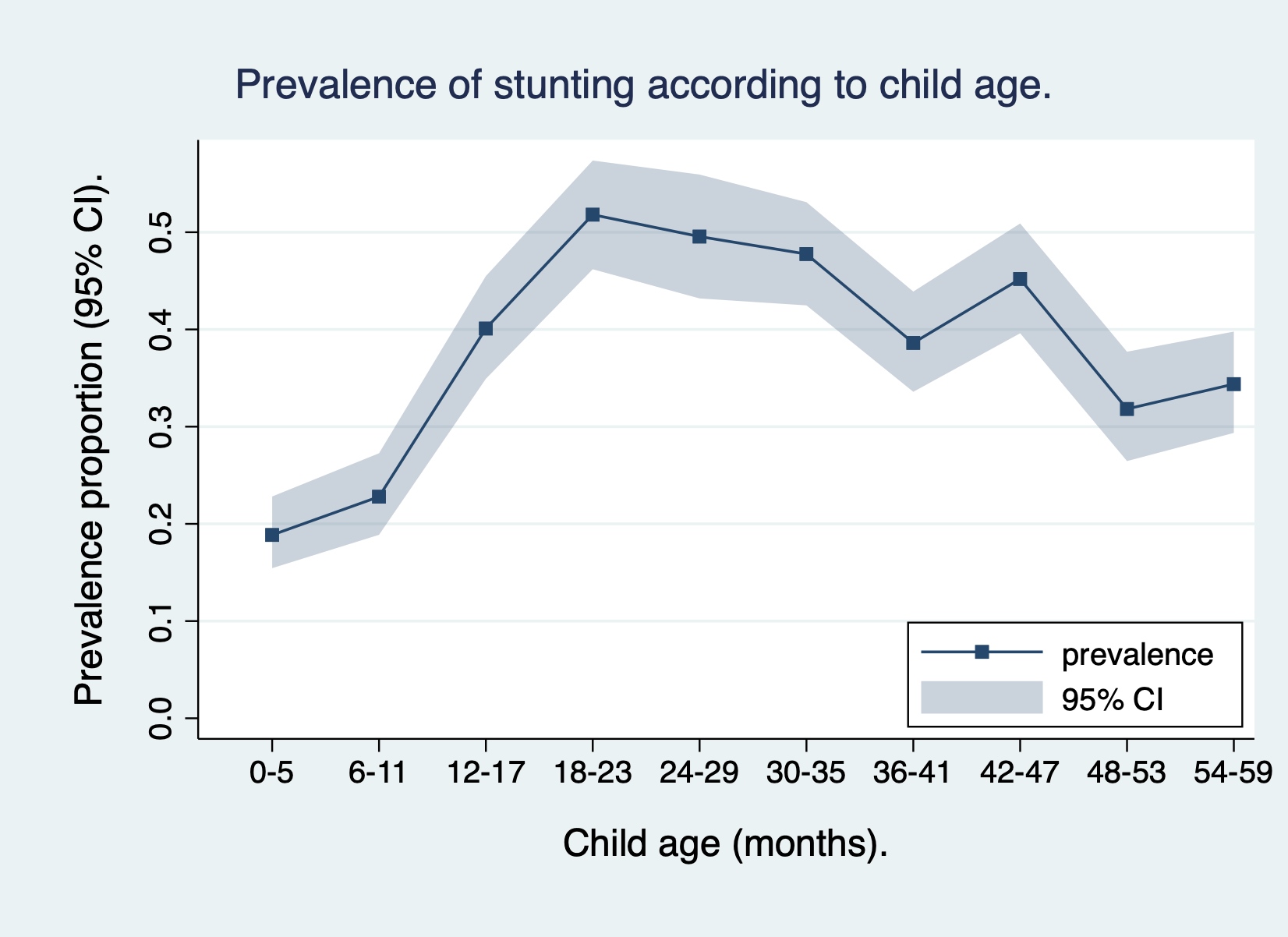

Supplement: S2 Fig — Graph shows the prevalence of stunting expressed as proportion with 95% confidence interval (95% CI), according to child age-group categorized in 6-month intervals. (TIF) [file pgph.0000983.s007.tif]
